# Small RNA and degradome sequencing used to elucidate the basis of tolerance to salinity and alkalinity in wheat

**DOI:** 10.1186/s12870-018-1415-1

**Published:** 2018-09-15

**Authors:** Huanan Han, Qi Wang, Lin Wei, Yu Liang, Jiulan Dai, Guangmin Xia, Shuwei Liu

**Affiliations:** 1 Key Laboratory of Plant Development and Stress Biology, Ministry of Education, School of Life Science, Shandong University, Qingdao, 266237 China; 2 Forest and Wetland Institute, Shandong Academy of Forestry, Jinan, 250014 China; 3 Environment Research Institute, Shandong University, Qingdao, 266237 China

**Keywords:** Alkalinity, Degradome, miRNA, Salinity, Small RNA, Wheat

## Abstract

**Background:**

Soil salinity and/or alkalinity impose a major constraint over crop yield and quality. An understanding of the molecular basis of the plant response to these stresses could inform the breeding of more tolerant varieties. The bread wheat cultivar SR3 exhibits an enhanced level of salinity tolerance, while SR4 is distinguished by its superior tolerance of alkalinity.

**Results:**

The small RNA and degradome sequencing was used to explore the miRNAs and corresponding targets associated with the superior stress tolerance of the SR lines. An examination of the small RNA content of these two closely related lines revealed the presence of 98 known and 219 novel miRNA sequences. Degradome libraries were constructed in order to identify the targets of the miRNAs, leading to the identification of 58 genes targeted by 26 of the known miRNAs and 549 targeted by 65 of the novel ones. The function of two of the stress-responsive miRNAs was explored using virus-induced gene silencing.

**Conclusions:**

This analysis indicated that regulation mediated by both auxin and epigenetic modification can be important in determining both salinity and alkalinity tolerance, while jasmonate signaling and carbohydrate metabolism are important for salinity tolerance, as is proton transport for alkalinity tolerance.

**Electronic supplementary material:**

The online version of this article (10.1186/s12870-018-1415-1) contains supplementary material, which is available to authorized users.

## Background

Soil salinity and alkalinity, either separately or jointly, represent a leading constraint over crop yield and quality, with up to 900 Mha of land classified as both saline and alkaline, about 340 Mha affected by just salinity and a further 560 Mha by just alkalinity [1]. The stress imposed on plants by combined alkalinity-salinity is distinct from that imposed by salinity in a neutral soil [2]. Elucidating the molecular basis of the plant response to saline/alkaline conditions could accelerate the breeding of crop varieties better able to tolerate these stresses. Much research effort has been expended on understanding the response to simple salinity [3]. With respect to alkalinity tolerance, apart from some evidence that the PKS5 mediated pathway is significant [4–7], the level of understanding of the response to this stress remains rather rudimentary.

Single-stranded RNA molecules ranging in length between 20 nt and 24 nt are referred to as microRNAs (miRNAs). Their biological significance lies in the observation that they negatively regulate up to 30% of eukaryotic genes, either through their guiding the cleavage of a complementary mRNA or via their inhibition of translation [8]. This mode of regulation acts in plants to modulate both development and the response to stress [9]. As an example, the abundance of the *Arabidopsis thaliana* miRNA miR393 responds to changes in the severity of a range of stress agents, including soil salinity, moisture deficiency and low temperature; its target is a component of the auxin signaling pathway [10]. Similarly, the abundance of miR398 is modulated by the oxidation status of plant tissue, thereby affecting the activity of target genes which encode scavengers of superoxide [11].

Wheat is one of the most important cereal in the world; its grain is processed into a large range of products which are consumed by at least 40% of the world’s population. The wheat plant is rather sensitive to both salinity and alkalinity, but conventional selection among the derivatives of an asymmetric somatic hybrid formed between the bread wheat cultivar Jinan 177 (JN177) and tall wheatgrass (*Thinopyrum ponticum*) has succeeded in identifying the two breeder’s lines Shanrong No. 3 and No. 4 (SR3 and SR4), with the former being more tolerant of salinity than JN177 [12, 13], and the latter more tolerant of alkalinity [14]. While SR3 out-performs SR4 in a neutral saline soil, the reverse is the case in an alkaline pH saline soil. Here, the focus was on their responses to both stresses, in particular through an analysis of miRNA expression. The analysis, based on miRNA and degradome sequencing, was able to identify a number of target genes, and has provided some insights into the ways in which the SR lines achieve their superior stress tolerance.

## Results

### Responses of wheat to salinity and alkalinity stress

**Fig. 1:**
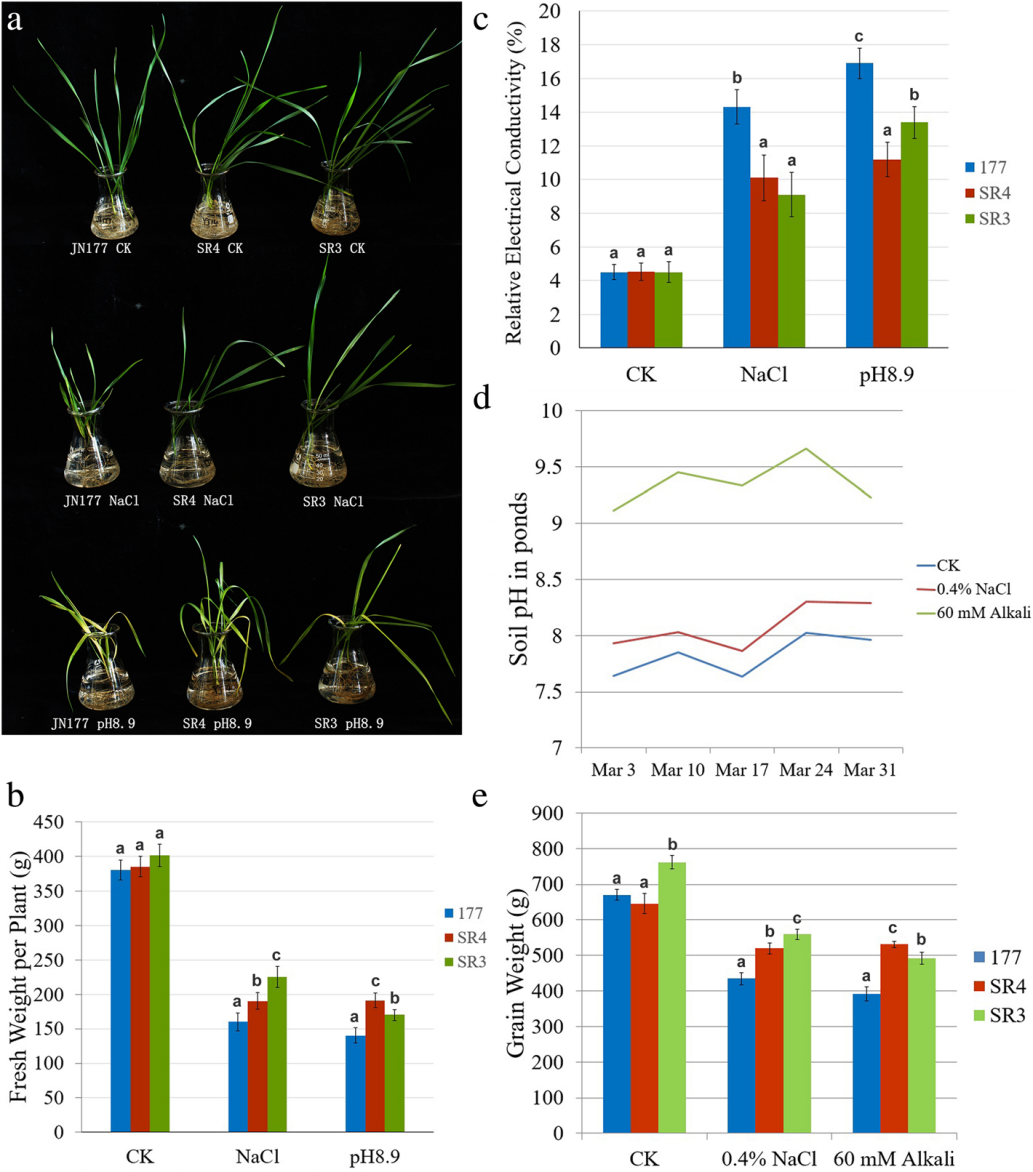
The selections SR3 and SR4 exhibit significant salinity and alkalinity tolerance. **a** The appearance of SR3, SR4 and their parental line JN177 following a week’s exposure to either 200 mM NaCl or 100 mM Na_2_CO_3_/NaHCO_3_. **b** Seedling fresh weights following growth under non-stressed and stressed conditions. **c** Leaf electrical conductivity of seedlings grown under non-stressed or stressed conditions. **d** Soil pH levels in the containers. **e** Grain yield of SR3, SR4 and JN177 plants grown in containers. Data given in the form mean ± s.d. (*n* = 3). a, b, c: means significant difference among samples, as determined by the One-way analysis of variance (ANOVA) test

When two-leaf-stage seedlings of JN177, SR3, and SR4 were exposed to either 200 mM NaCl or 100 mM Na_2_CO_3_/NaHCO_3_ for seven days, both SR lines tolerated more effectively than did JN177 to both stresses; moreover, SR3 exhibited a higher tolerance of salinity than SR4, while SR4 showed more tolerant to alkalinity than SR3 (Fig. 1 A,B). The same behavior was apparent when tolerance was assessed in terms of the electrical conductivity of the leaf (Fig. 1C). The three lines were subsequently grown under irrigation with either fresh water, saline water (0.4% NaCl) or alkaline water (60 mM Na_2_CO_3_/NaHCO_3_); the soil pH in the three containers was, respectively 7.5–8.0, 7.8–8.3 and 9.3–9.8 (Fig. 1D). SR3 and SR4 plants both yielded better than JN177 plants in the presence of either stress, but SR3 out-yielded SR4 in the saline conditions and vice versa in the alkaline ones (Fig. 1E). The reactive oxygen species (ROS) of the three wheat lines were also measured. The results indicated that SR3 plants experiencing salinity stress accumulated a much higher level of ROS than did either JN177 or SR4 plants, while under alkalinity stress, SR4 plants were better able to restrict the level of ROS than either JN177 or SR3 ones (Additional file 1: Figure S1).

### The acquisition of miRNA sequence

**Fig. 2:**
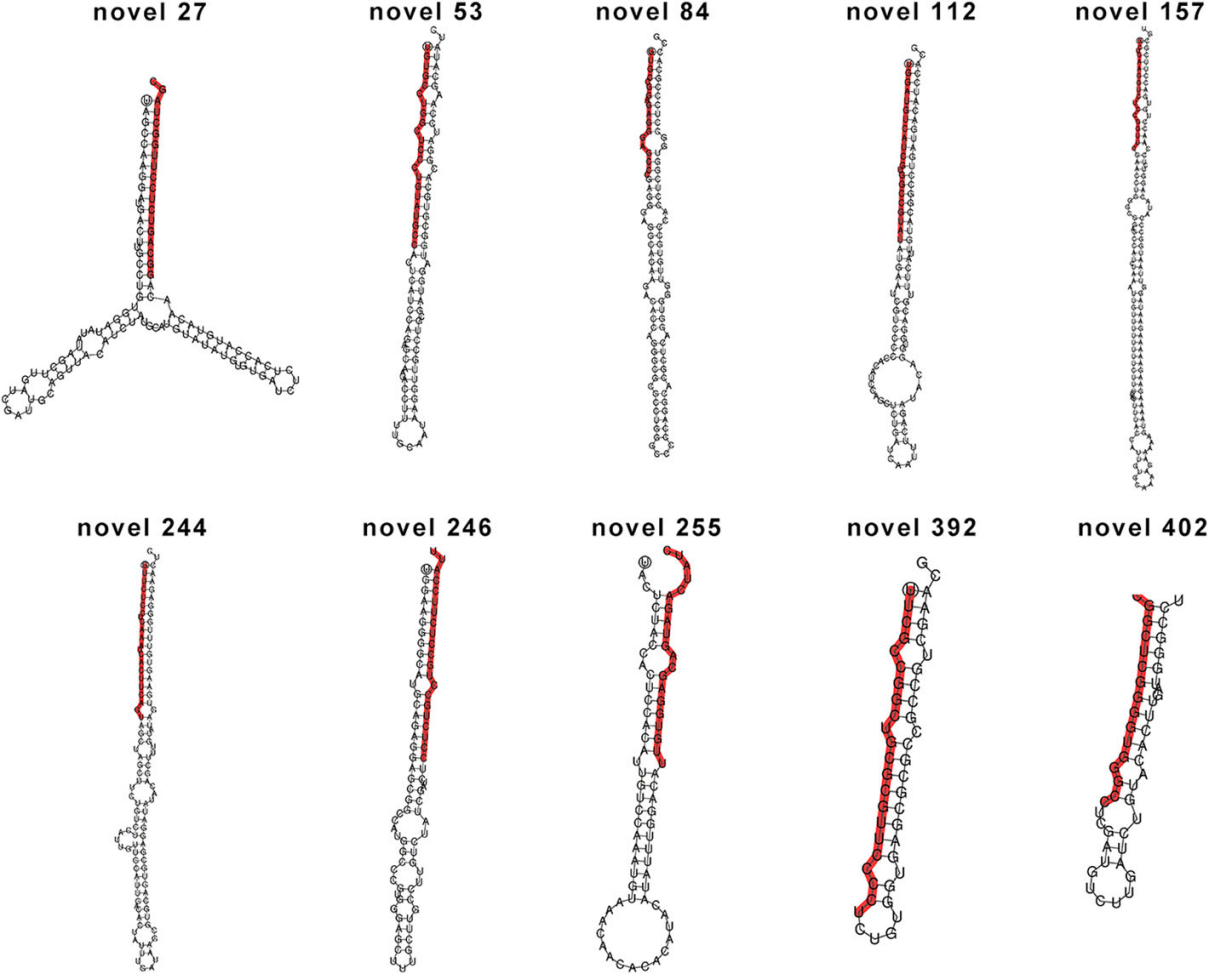
Secondary structure prediction of ten novel wheat miRNA precursors. The red colored sequences represent mature miRNA

**Fig. 3:**
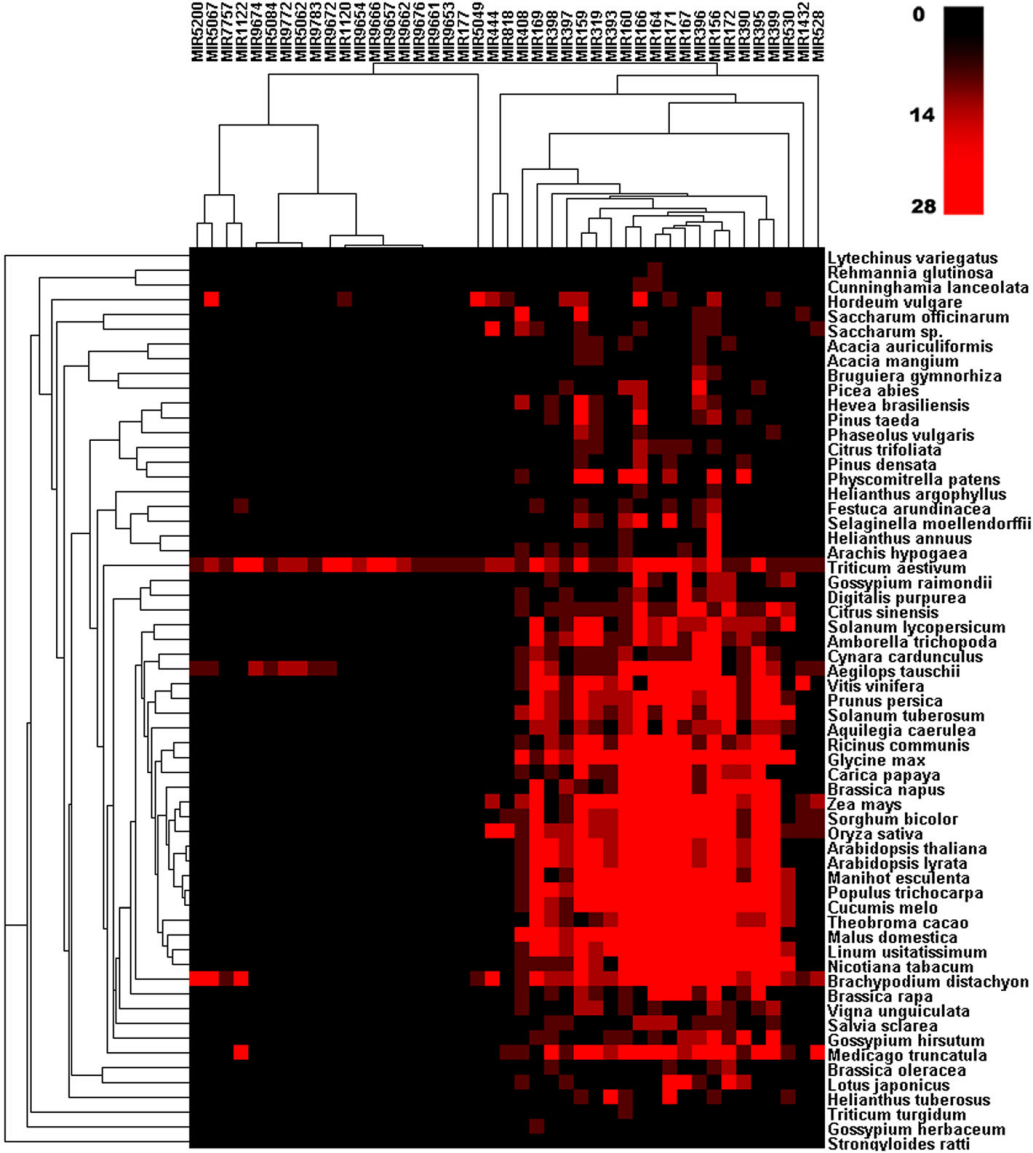
Phylogenetic analysis of miRNAs extracted from wheat. The sequences were aligned to other plant miRNAs, and a heatmap assembled to demonstrate the relationship of the wheat miRNAs to those of other plant species. The intensity of the red color show positive correlation with the number of relative miRNA family members

Nine small RNA libraries were constructed from RNA extracted from the roots of JN177, SR3 and SR4 seedlings exposed to either no stress, salinity stress or alkalinity stress. After removal of low quality sequences, adapters and polyA/T/G/C sequences, each library yielded 12–22 million reads (Table 1). Of these between 5 and 13 million were in the size class 18–30 nt. The most abundant size was 24 nt in each case (Additional file 2: Figure S2). When the sequences were aligned with wheat unigene and wheat genomic sequence using the BLAST algorithm, 50–70% of them perfectly matched to wheat genome which were mostly “+”Mapped sRNA (Table 1). The aligned sequences were then categorized by reference to the Rfam database (Additional file 3: Figure S3), which allowed for the removal of rRNA, tRNA, snRNA and snoRNA sequences; the remaining reads were then used to query the set of wheat miRNAs assembled in miRBase release 21 (www.mirbase.org), applying a strict alignment criterion (zero mismatches). This process yielded a set of 85 already documented miRNAs (“known” miRNAs), 49 of which were associated with 27 families (Additional file 4: Table S1, Additional file 5: Table S2). The remaining reads were analyzed using miREvo [15] and mirdeep2 [16] software: this analysis identified 219 of them as bona fide miRNAs (“novel” miRNAs) (Additional file 6: Table S3). The stem-loop secondary structure of ten novel miRNAs is shown in Fig. 2. Aligning the latter sequences with miRNAs documented in other species (the alignment criterion was relaxed to allow for up to two mismatches) resulted in the recognition of 35 sequences which were homologous with 24 non-wheat miRNAs (Additional file 7: Table S4). About one half of these miRNAs were relatively abundant (mean abundance of > 10 across the nine libraries) (Additional file 8: Table S5). There was a pronounced bias in both the known and novel miRNAs for A as the mature sequence’s first base (Additional file 9: Figure S4). Many of the mature sequences generated from the set of known miRNAs featured a G in the middle and a C at their 3′ end (Additional file 10: Figure S5). On the basis of an evolution conservative analysis, 43 families were identified, among which 23 families were represented in several plant species, while the other 20 families were represented solely in wheat or in a small number of wheat-related species (Fig. 3).

**Table 1:** Statistics of sRNA in nine small RNA libraries

Sample	Total_reads	Clean reads	Total sRNA	Mapped sRNA	“+”Mapped sRNA	“-”Mapped sRNA
JNC	13905227	13622140	8570198	5815655 (67.86%)	5572036	243619
SR4C	23460206	22745078	13442946	9491485 (70.61%)	9083128	408357
SR3C	13957096	13189289	6137061	3083944 (50.25%)	2863890	220054
JNS	12620549	12028013	5683955	3109524 (54.71%)	2905578	203946
SR4S	12666276	12051099	5002942	2804316 (56.05%)	2627315	177001
SR3S	19071353	18115112	6947715	3928870 (56.55%)	3682891	245979
JNA	15807924	15080336	7467791	3783971 (50.67%)	3541206	242765
SR4A	14615338	13555241	5385320	2770732 (51.45%)	2601734	168998
SR3A	14899916	14142849	6718130	3350157 (49.87%)	3162281	187876

Note: “JN” means samples of JN177. “C” in JNC, SR3C, SR4C means control condition, and “S”, “A” means saline or alkaline treatment, respectively

### The abundance of some miRNAs was influenced by salinity and/or alkalinity stress

**Fig. 4:**
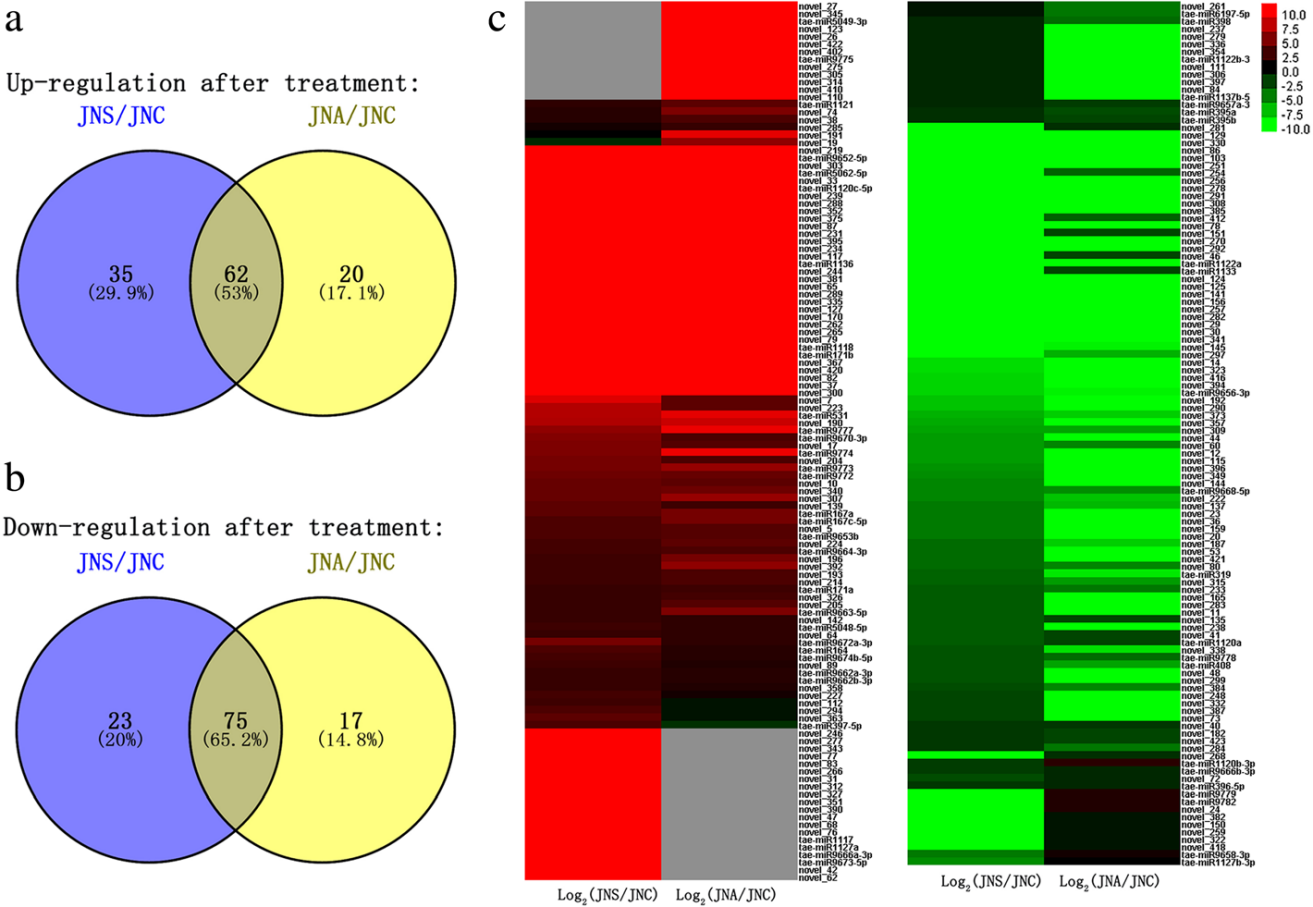
The abundance of miRNAs in the roots of wheat plants JN177 exposed to salinity or alkalinity stress. miRNAs which (**a**) increased, (**b**) decreased in abundance in response to stress. **c** A heatmap detailing the altered abundance of JN177 miRNAs in plants subjected to salinity or alkalinity stress. The left part was for stress induced miRNA while the right part was for stress repressed ones, miRNAs which increased in abundance are marked in red, and those which decreased in green. Gray color indicate the non-detection of the relevant miRNA

**Fig. 5:**
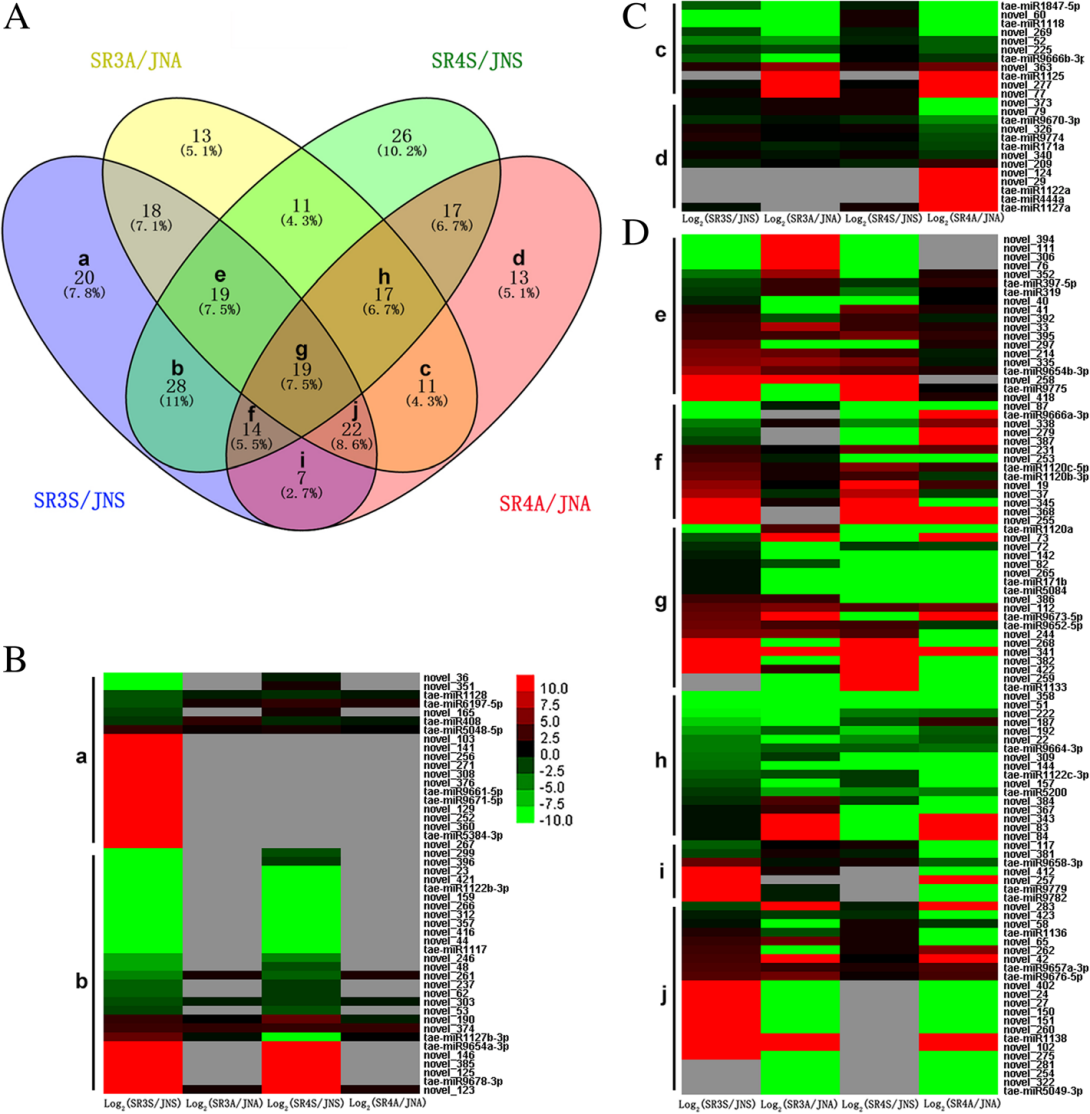
The abundance of miRNAs in roots of introgression lines which have changed expression levels comparing with JN177 under stress treatment. **A** Differences in abundance between introgression line and JN177 plants subjected to salinity or alkalinity stress. **B-D** A heatmap showing the relative abundance of miRNAs in SR3/SR4 compared to JN177 plants exposed to either salinity or alkalinity stress. **B** miRNAs which were up or down regulated in SR3 only under saline stress; **C** miRNAs which has different expression levels in SR4 when specially treated by alkali stress; **D** miRNAs expressed differently in introgression lines under either saline or alkaline stress conditions. As described above, the red stood for increased abundance and the green for decreased ones. Gray means non-detection

**Fig. 6:**
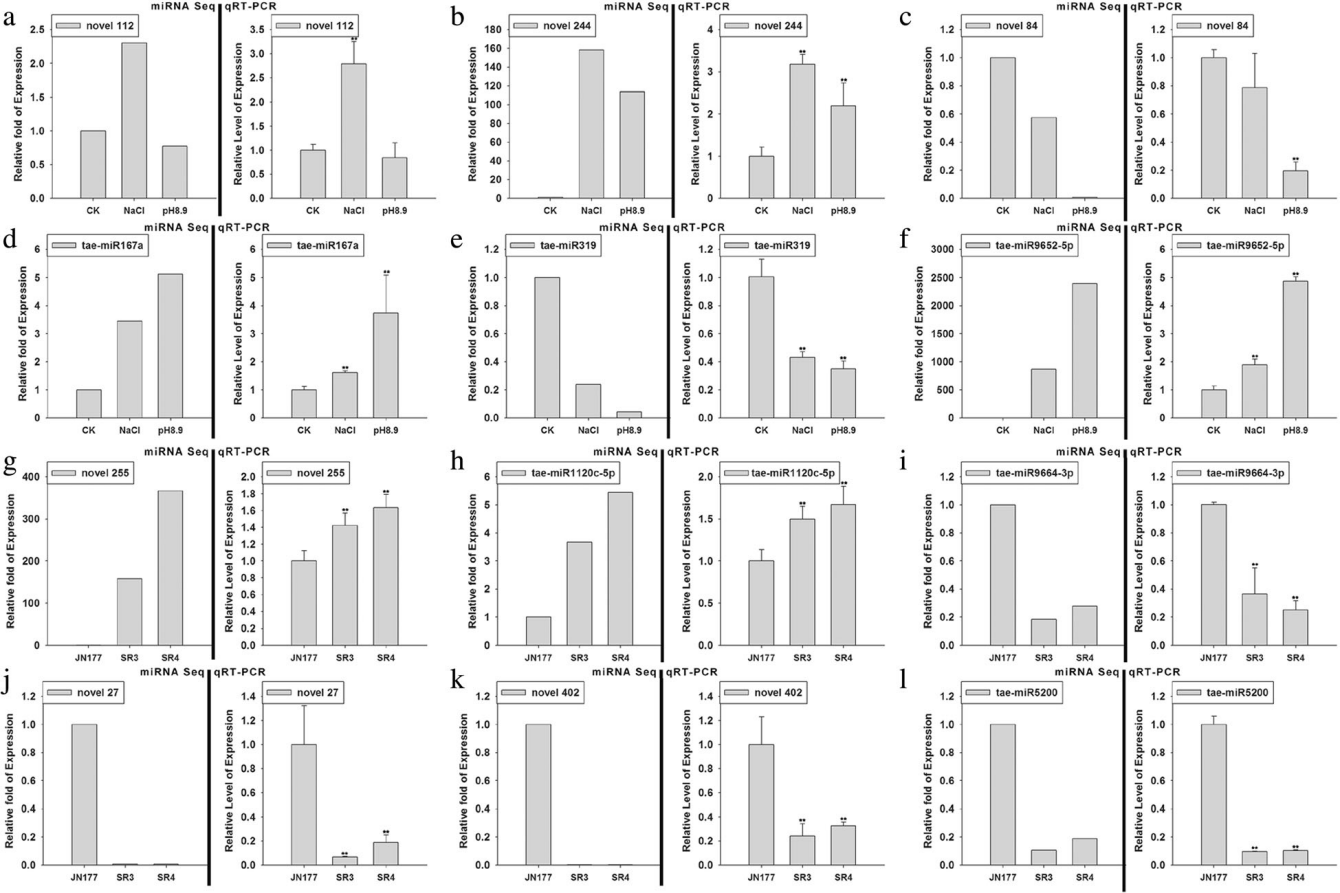
The abundance of a selection of 12 stress-responsive miRNAs assayed using qRT-PCR. **a-f** Relative abundance of the miRNAs in JN177 plants exposed to stress. **g-i** Relative abundance of the miRNAs in SR3/SR4 plants compared to JN177 plants exposed to salinity stress. **j-l** Relative abundance of the miRNAs in SR3/SR4 plants compared to JN177 plants exposed to alkalinity stress. The left hand histogram shows abundances as estimated from miRNA sequencing, and the right hand one as estimated by qRT-PCR. The latter data are given in the form mean ± s.d. (*n* = 3). CK: non-stressed conditions, NaCl: salinity stress conditions, pH 8.9: alkalinity stress conditions. *, **: means differed significantly at, respectively, *P* < 0.05 and < 0.01, as determined by the Student’s *t*-test

Having normalized the abundance of the miRNAs in the form of transcripts per million (TPM), a selection was made, based on a TPM threshold of 10 in at least one library. Under salinity stress, the expression level of a total of 97 miRNAs were up-regulated while 98 down-regulated in JN177; in contrast, the abundance of 82 miRNAs were increased while 92 decreased in JN177 under alkalinity; in all these stress responsive miRNAs, 62 miRNAs were up-regulated and 75 down-regulated in JN177 under both salinity and alkalinity stresses (Fig. 4, Additional file 11: Table S6). When the responses of SR3 and JN177 were compared, 147 miRNAs proved to be differentially abundant in plants exposed to salinity and 130 in those exposed to alkalinity. The equivalent numbers derived from the comparison of SR4 and JN177 were, respectively, 151 and 120 (Fig. 5, Additional file 12: Table S7). When the abundance of 12 of the miRNAs was checked using quantitative real-time PCR (qRT-PCR), the outcomes were fully consistent with the sequencing-based data (Fig. 6).

### Targets of salinity/alkalinity stress-related miRNAs validated by degradome sequencing

**Fig. 7:**
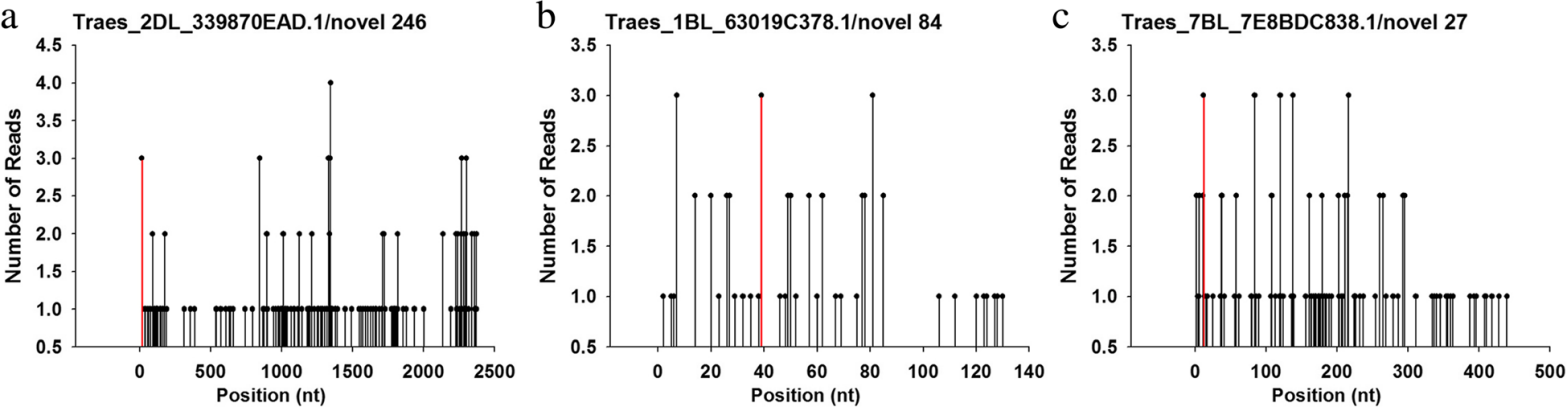
The validation of miRNA targets based on degradome sequencing. The target plots show sequence abundances (read counts) throughout the length of the indicated transcripts. The red lines in the t-plot indicate sequence reads consistent with miRNA-directed cleavage. **a** The target of novel 246. **b** The target of novel 84. **c** The target of novel 27

**Fig. 8:**
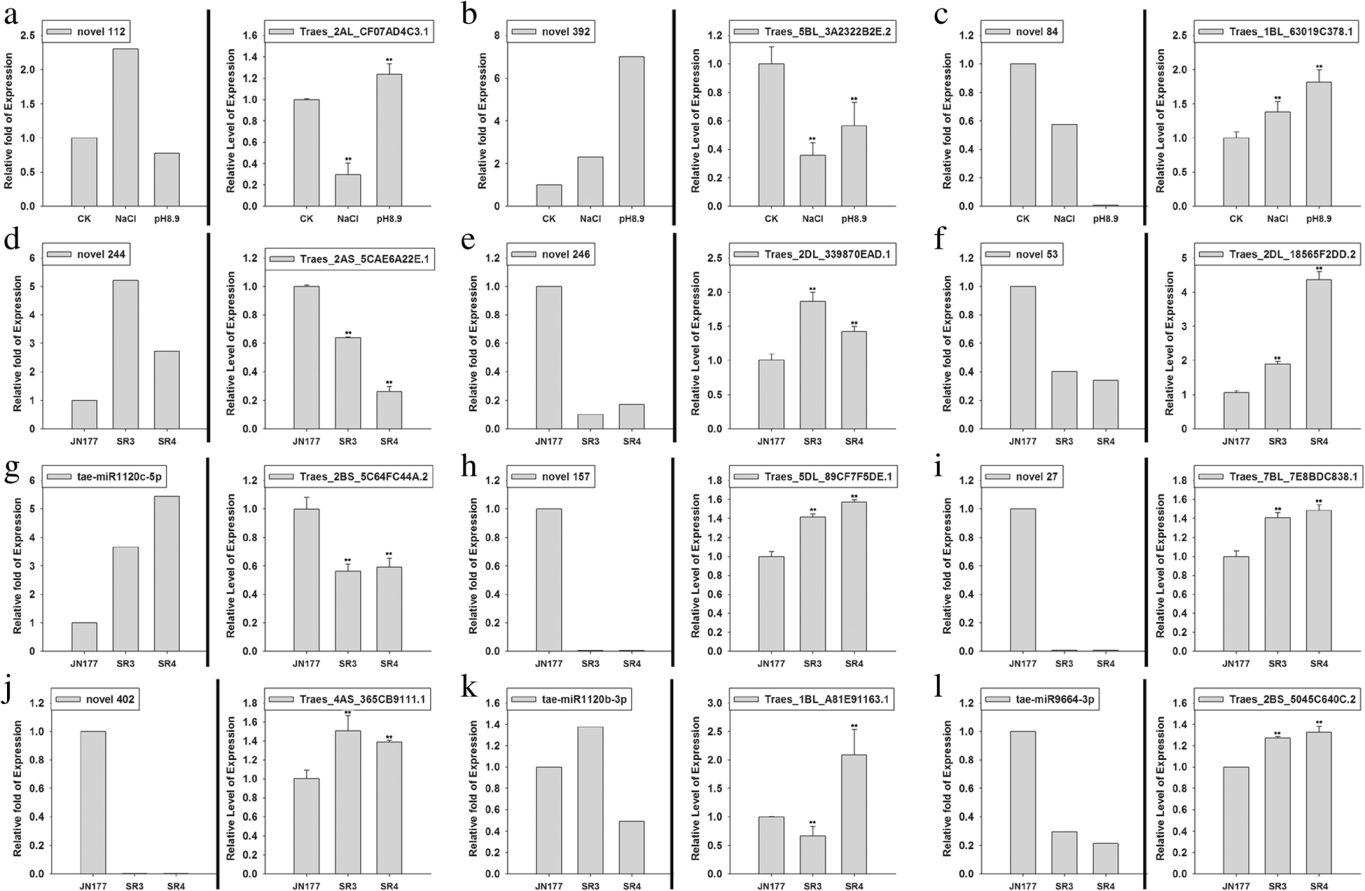
The relationship between miRNAs and their target genes. The abundance of target gene transcripts present in the degradome were detected by qRT-PCR and compared with the abundances of the relevant miRNA obtained from the RNA sequencing data. **a-c** miRNA/target pairs involving JN177 genes responsive to stress. **d-g** miRNA/target pairs which were differentially abundant in plants exposed to salinity stress. **h-l** miRNA/target pairs which were differentially abundant in plants exposed to alkalinity stress. For each pair, the left hand histogram shows miRNA abundance as estimated from the sequence data, and the right hand one the target’s transcript abundance. qRT-PCR data are given in the form mean ± s.d. (*n* = 3). *, **: means differed significantly at, respectively, P < 0.05 and < 0.01, as determined by the Student’s *t*-test

**Fig. 9:**
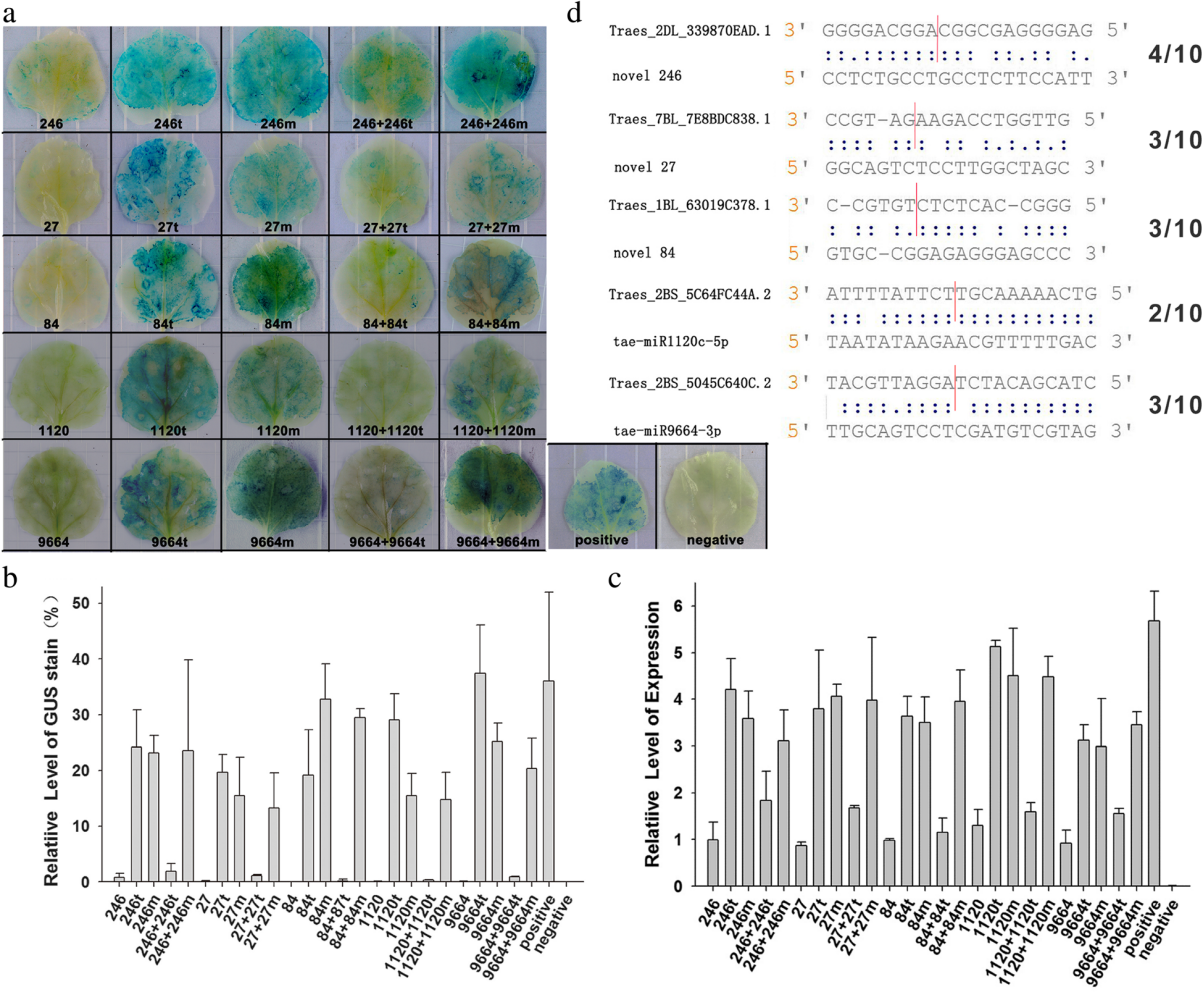
*In planta* visualization of the cleavage of target genes by miRNAs in tobacco leaves. **a** GUS activity associated with a miRNA/target pair. miRNAs or cleavage contiguous fragments of target genes were cloned to down-stream of CaMV35S promoter in pStart-GUS vector. For each miRNA/target pair, the GUS stain assay was made by five parts, including miRNA (marked by 246, 27, 84, 1120 or 9664, indicating novel_246, novel_27, novel_84, miR1120c and miR9664, respectively), target fragment (marked by 246 t, 27 t, 84 t, 1120 t or 9664 t), mutant target fragment (marked by 246 m, 27 m, 84 m, 1120 m or 9664 m), co-expressed miRNA/target (marked by 246 + 246 t, 27 + 27 t, 84 + 84 t, 1120 + 1120 t or 9664 + 9664 t) and co-expressed miRNA/mutant target (246 + 246 m, 27 + 27 m, 84 + 84 m, 1120 + 1120 m or 9664 + 9664 m). The pStart-GUS vector was used as the positive control, while a wild type tobacco leaf served the negative control. **b** Quantification of GUS activity in transformed tobacco leaves calculated from mean pixel densities. **c** Relative expression level of GUS gene in transformed tobacco leaves with a miRNA/target pair. **d** RLM 5’-RACE to validate the target mRNA cleavage sites for five miRNAs. The red line show the cleavage sites by miRNA. 10 clones were sequenced after RLM 5’-RACE for every miRNA/Target pair and the number of sequences found at the exact cleavage site was showed

An attempt was made to identify miRNA targets using target prediction combined with degradome sequencing. The outcome of the former approach was the identification of 296 predicted targets for 65 of the miRNAs (Additional file 13: Table S8). Two degradome libraries were constructed separately, with the first library from roots of JN177 while the second from a bulk of SR3 and SR4. In all, respectively 18.6 and 13.6 million tags were identified, of which, respectively, 5.4 and 4.1 million were mapped onto a wheat unigene sequence (Table 2). The net result was the identification of 58 target genes for 26 of the known miRNAs and of 549 target genes for the 65 novel miRNAs (Fig. 7, Additional file 14: Table S9). To investigate the effect of miRNAs on their targets, the transcription profile of the putative targets of 12 of the stress-responsive miRNAs were derived using qRT-PCR (Fig. 8). This analysis showed that the genes *Taes_2AL_CF07AD4C3.1* and *Taes_5BL_3A2322B2E.2* were both down-regulated by the combined stress, while the abundance of the two cognate miRNAs novel_112 and novel_392 was enhanced by the stress treatment. As a result of the stress treatment, the gene *Taes_1BL_63019C378.1* was up-regulated, while the abundance of its corresponding miRNA novel_84 was decreased. The abundance of a further nine miRNAs was shown as negatively correlated with that of their corresponding target in the contrast SR3/SR4 vs JN177 plants exposed to either salinity or alkalinity. A selection of five of the targets of the stress-induced miRNAs was made for an *in planta* assay in tobacco (Additional file 15: Figure S6). The target genes were *Traes_2DL_339870EAD.1* (novel_246), *Traes_7BL_7E8BDC838.1* (novel_27), *Traes_1BL_63019C378.1* (novel_84), *Traes_2BS_5C64FC44A.2* (tae-miR1120c) and Traes_2BS_5045C640C.2 (tae-miR9664). Each was fused to *GUS* and expressed transiently in tobacco. In every case, the GUS signal strength was reduced when the target gene was co-expressed with its corresponding miRNA (Fig. 9a, b). The expression levels of GUS gene showed the similar pattern as GUS signal strength (Fig. 9c). 5’RACE assay was also performed to confirm the cleavage of target sequences in tobacco and the results coincide with the degradome sequencing data (Fig. 9d).

**Table 2:** Read statistics of two degradome libraries

sRNA categories	Library I		Library II	
	Taq numbers	Unique taqs	Taq numbers	Unique taqs
rRNA	5100020 (27.42%)	68908 (0.94%)	3873416 (28.39%)	63786 (1.13%)
tRNA	100 (0.00%)	38 (0.00%)	68 (0.00%)	34 (0.00%)
snRNA	88 (0.00%)	55 (0.00%)	64 (0.00%)	50 (0.00%)
snoRNA	787 (0.00%)	165 (0.00%)	548 (0.00%)	148 (0.00%)
polyN	100640 (0.54%)	77381 (1.05%)	52561 (0.39%)	37500 (0.67%)
cDNA_sense	5399536 (29.03%)	2934822(39.96%)	4135477 (30.31%)	2393485 (42.46%)
cDNA_antisense	160933 (0.87%)	70518 (0.96%)	52400 (0.38%)	28006 (0.50%)
Other	7837485 (42.14%)	4193190 (57.09%)	5528745 (40.52%)	3113910 (55.24%)
Total	18599589	7345077	13643279	5636919

Note: Library I was for JN177 and library II was a mixed library composed of SR3 and SR4

### Functional analysis of target genes

Both the Gene Ontology (GO) and Kyoto Encyclopedia of Genes and Genomes (KEGG) databases were used to assign function to the genes identified as miRNA targets. According to the GO analysis, those which were classified as being salinity-responsive encoded products were involved in two categories of molecular function and eight categories of biological process: the most abundant term associated with molecular function was “binding”, and the most abundant associated with biological process was “regulation of biological process”. With respect to the alkalinity-responsive genes, four categories of molecular function and eight of biological process were identified, with the most abundant terms for the former being “transporter activity” and for the latter “regulation of biological process” (Additional file 16: Figure S7). According to KEGG analysis, the salinity-responsive genes were enriched with respect to 25 pathways including “one carbon pool by folate”, “phenylalanine metabolism”, “sphingolipid metabolism”, “glycerophospholipid metabolism” and “glycerolipid metabolism”; the set of alkalinity-responsive ones were similarly enriched with respect to 25 pathways, which featured “phenylalanine metabolism”, “glutathione metabolism”, “ubiquinone and other terpenoid-quinone biosynthesis” and “oxidative phosphorylation” (Additional file 17: Table S10).

### Salinity- and alkalinity-responsive miRNA targets

The target genes responsive to both salinity and alkalinity in JN177 were functionally diverse. A group of 14 genes was associated with signal transduction and a second group of 28 with transcription regulation (including genes encoding various protein kinase, splicing factors and transcription factors such as MYB, NAC, WRKY and bZIP). One target of novel_367 is a bZIP60-like protein, while bZIP60 was related to ER stress response and associated to salt stress in *Arabidopsis thaliana* [17]. WRKY57 was one target of novel_300, which was reported to play role in regulating drought tolerance [18]. A group of 23 genes encoded metabolic proteins, involving carbohydrate, lipid and phenylpropanoid metabolism. For example, two targets of novel_367, encoding glycosyltransferase and sucrose-phosphate synthase, were key enzymes in carbohydrate metabolism process. A lipid metabolism associated lipid-transfer gene, was a target of novel_190. A fourth group comprised seven genes involved in epigenetic modification, encoding histone variants, histone acetyltransferase and chromatin structure remodeling factors (targets of novel_223, novel_284, novel_53 or novel_190). Two genes associated with auxin synthesis (one encoding an indole-3-pyruvate monooxygenase and the other flavin-containing monooxygenase 1) were also identified (Targets of novel_300). Among the JN177 genes responsive to salinity but not alkalinity were three encoding components of the jasmonate (JA) signaling pathway (allene oxide cyclase and two TIFY transcription factors, as targets of novel_246 or novel_390) and five encoding transporters (a CMP-sialic acid transporter, an ABC transporter, a polyamine transporter, a nitrate transporter and an aquaporin). Among the JN177 genes responsive to alkalinity but not salinity were one encoding 2-oxoglutarate-dependent dioxygenase (auxin catabolism, target of novel_402) and three encoding components of lipid metabolism (phospholipid-transporting ATPase, ω-3 fatty acid, phosphatidylglycerol phosphatidylinositol transferase, targets of novel_354, novel_410, novel_314) (Additional file 18: Table S11).

A comparison of miRNA abundances between JN177, SR3 and SR4 plants subjected to either salinity or alkalinity stress revealed that in SR3, 69 miRNAs were differentially abundant from JN177 only under salinity stress, while 52 and 78 miRNAs showed differential abundance under alkalinity stress or under both stresses. In SR4, respectively, 84, 53 and 67 miRNAs were differentially abundant in comparison to JN177 under salinity, alkalinity or both stresses (Fig. 5). Although both SR3 and SR4 are salinity and alkalinity tolerant, SR3 was superior for neutral salt while SR4 superior for alkali salt stress, thus, the 48 (28 + 20) salinity- or 24 (11 + 13) alkalinity-specific differentially expressed miRNAs might closely associated with salinity or alkalinity stress tolerance of the two wheats (Fig. 5).

The targets of the miRNAs which became differentially abundant as a result of salinity stress included seven involved in signal transduction, seven in transcription regulation and six in carbohydrate metabolism. The signal transduction-associated genes comprised one encoding the calcium-binding protein CML13 (targets of novel_246), one encoding a rac-like GTP-binding protein (targets of novel_190) and five encoding a protein kinase (targets of novel_190, novel_312 or tae-miR9678-3p). Each of the transcription regulation-associated genes encoded a transcription factor (two MYB and two bZIP, targets of novel_190 or novel_246). The carbohydrate metabolism-associated genes comprised one encoding β-D-xylosidase (novel_190), one encoding α-mannosidase (novel 421), one encoding α, α-trehalose phosphate synthase (novel_312) and two encoding pectin synthesis (novel_190 or novel_374). A further target encoded a TIFY transcription factor associated with JA signaling (novel_246), along with a gene encoding a polyamine transporter (novel_246) and two encoding a peroxidase (novel_190) (Additional file 19: Table S12). The targets of the miRNAs which became differentially abundant as a result of alkalinity stress comprised six genes associated with reactive oxygen species (ROS) homeostasis, namely four encoding a respiratory burst oxidase homolog (tae-miR1122a) and two α sub-complex subunits of NADH dehydrogenase 1 (novel_52). One of the two genes associated with phospholipid metabolism encoded phosphoinositide phosphatase and the other glycerophosphodiester phosphodiesterase (tae-miR1127a or tae-miR171a). Finally, there were two genes encoding a histone variant (H2A.Z, targets of novel_52) and one a vacuolar H^+^ adenosine 5′-triphosphatase (ATPase) (tae-miR9666b-3p) (Additional file 20: Table S13).

### Functional analysis of two differentially abundant miRNAs using virus-induced gene silencing (VIGS)

**Fig. 10:**
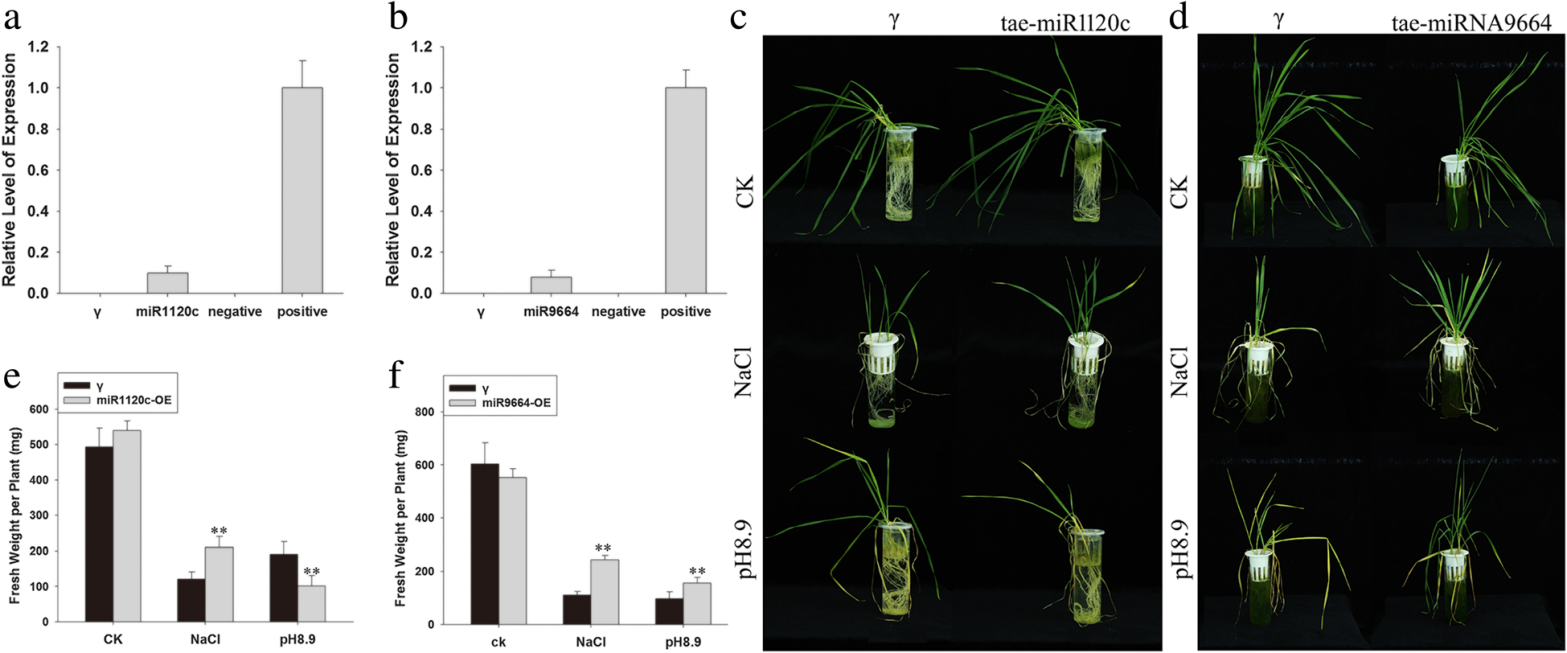
The use of VIGS to confirm the function of two miRNAs (miR1120c and miR9664) which were altered in abundance in response to abiotic stress. **a-c** miR1120c, **d-f** miR9664. **a**, **d** The abundance of the miRNA in VIGS transformed wheat seedlings as assessed using qRT-PCR. The VIGS γ vector (marked “γ”) was used as a vector control and the pri-miRNAs were recombined within the γ vector to achieve their over-expression. The recombined miRNA-γ plasmids were used as a positive control, and the absence of any template as a negative one. **b**, **e** The phenotype of wheat seedlings over-expressing miR1120c or miR9664 in the presence of either salinity or alkalinity stress. **c**, **f** The fresh weight of the seedlings illustrated in (**b**) and (**e**), respectively. The values shown represent the mean of ten seedlings per line, and are given in the form mean ± s.d. (*n* = 3). *, **: means differed significantly at, respectively, P < 0.05 and < 0.01, as determined by the Student’s *t*-test

The sequences of two of the differentially abundant miRNAs (tae-miR1120c and tae-miR9664) were transiently over-expressed in wheat seedlings using the VIGS method (Additional file 15: Figure S6). There was phenotypic effect of either transgene when the plants were grown in the absence of stress. However, in the presence of salinity stress, over-expressors of tae-miR1120c were more tolerant than plants carrying only an empty vector, but were less tolerant of alkalinity. In contrast, the tae-miR9664 over-expressors displayed an improved tolerance to both stresses (Fig. 10). The patterns of stress tolerance were consistent with the abundance of these two miRNAs in SR3 and SR4 plants subjected to stress (Additional file 21: Figure S8).

## Discussion

Wheat yields are compromised on soils affected by either salinity and/or alkalinity, which underlines the importance of identifying sources of tolerance such as represented by the introgression lines SR3 and SR4. These lines provide a useful platform for gaining an understanding of the mechanistic basis of salinity and alkalinity tolerance, since they are closely related both to one another and to their common, stress-intolerant parent JN177. The recognition that miRNAs are an important component of the plant response to stress is relatively recent [19–21]; however, until now, there has been very little experimental evidence generated to demonstrate which specific miRNAs are involved in the determination of either salinity and/or alkalinity tolerance in wheat.

### Auxin is important for the expression of both salinity and alkalinity tolerance

The phytohormone auxin has long been known to be intimately involved in controlling both plant growth and development, but more recently it has become apparent that it is also important in the context of the abiotic stress response. Under salinity stress, the activation of Na^+^/H^+^ antiporters is known to destabilize the cellular pH environment, which interferes with the transport of auxin [22]. The over-expression *YUCCA* (the product of which is associated with auxin synthesis) tends to enhance drought tolerance [23, 24]. Auxin regulates the activity of H^+^-ATPase [25], so a loss-of-function of the gene *PIN2,* which encodes an auxin efflux carrier, results in a fall in H^+^-ATPase activity and a greater sensitivity to alkalinity stress [26]. The involvement of miRNAs in the regulation of auxin signaling has been shown repeatedly. Thus, for example, in *A. thaliana* plants subjected to salinity stress, the abundance of miR393 rises, resulting in the repression of its target gene *AFB2*, which encodes an auxin-responsive factor [10, 27]. ARFs are known to be either directly or indirectly regulated by miRNAs [28–30]. Here the abundance of the miRNA novel_300 was shown to be responsive to both salinity and alkalinity stress; the degradome analysis implied that its targets include two genes encoding products associated with auxin synthesis, namely YUCCA10 and a flavin-containing monooxygenase 1. Given that a rise in tissue auxin content typically enhances the level of abiotic stress tolerance [23, 24], the conclusion is that novel_300 mediated auxin synthesis may well be involved in determining both the salinity and alkalinity tolerance of wheat.

### Both salinity and alkalinity tolerance are in part epigenetically regulated

The rapid creation of a reversible epigenetic modification provides an important means for the plant to regulate its transcriptome in a way which helps it survive the stress. DNA methylation is known to be rapidly induced by salinity stress, in a way which regulates the expression of a number of salinity responsive genes [31–33]. For example, the level of methylation affecting the promoter regions of four soybean transcription factors has been shown to be sensitive to salinity stress [34]. The somatic hybridization process used to generate the SR lines induces large-scale changes in the genome’s cytosine methylation profile [35], and such changes occurring in promoter sequences can affect the activity of a number of salinity-responsive genes [36]. The differential transcription of at least eight genes in SR4 (compared to JN177) plants experiencing alkalinity stress has been shown to be driven by epiallelic variation [14]. Here, the abundance of the miRNAs novel_190 and novel_223 was increased by stress, while that of novel_53 and novel_284 was reduced, and six of their targets experienced epigenetic modification (Additional file 18: Table S11). Provisionally at least the conclusion is that epigenetic modification plays a part in determining the abiotic response.

### ROS homeostasis is required for both salinity and alkalinity tolerance

Both salinity and alkalinity can induce the production of ROS, which if not controlled, can ultimately trigger cell death. Thus it was very important for plants to ensure cellular ROS homeostasis, which was the result of a balance between ROS production (produced from disturbed respiration or photosynthesis) and ROS scavenging (usually associated with antioxidase such as SOD, POD, CAT, APX or antioxidants such as proline). The product of the ROS responsive gene *TaSRO1* is a key component of the enhanced salinity tolerance exhibited by SR3, achieved partly through its regulation of ROS homeostasis and partly through its contribution towards maintaining DNA integrity [37]. SR3 plants experiencing salinity stress accumulated a much higher level of ROS than did either JN177 or SR4 plants, while under alkalinity stress, SR4 plants were better able to restrict the level of ROS than either JN177 or SR3 ones (Additional file 1: Figure S1). The abundance of novel_52 was repressed significantly in salinity-stressed SR3 but only slightly repressed in salinity-stressed SR4 (Additional file 12: Table S7); the targets of this miRNA include two α sub-complex subunits of NADH dehydrogenase 1, which involved in the generation of ROS. Thus, the de-repression of NADH dehydrogenase 1 might enhance the ROS level of SR3 which was reported to be responsible for its salinity tolerance [37]. A transcriptomic analysis has revealed a few genes encoding ROS scavenging enzymes to be more abundantly transcribed in alkalinity-stressed SR4 plants compared to JN177 ones [14]. Here, it was apparent that the abundance of tae-miR1122a was enhanced in alkalinity-stressed SR4 plants; the targets of this miRNA include four genes encoding a respiratory burst oxidase homolog (*Rboh*), proteins which are intimately involved in the generation of ROS; the proposition is that in alkalinity-stressed SR4 plants, tae-miR1122a represses the production of ROS by its targeting of *Rboh* genes. A suppression of ROS production, along with an enhanced ROS scavenging ability allowed SR4 to limit the accumulation of ROS in the presence of alkalinity stress. The proposition is that the enhanced salinity tolerance of SR3 was in part due to its ability to regulate ROS homeostasis and maintain DNA integrity, while the enhanced alkalinity tolerance of SR4 was related to its ability to avoid excessive ROS accumulation.

### JA signaling contributes to salinity tolerance

JA is a vital signaling molecule across the plant kingdom, and is known to participate in the abiotic stress response [38–40]. JA synthesis relies on the enzyme allene oxide cyclase (AOC), and it has been observed previously that the wheat gene *TaAOC1* can be induced in SR3 plants by exposing them to either salinity or drought stress; meanwhile its constitutive expression in both wheat and *A. thaliana* enhances the plants’ salinity tolerance [41]. Here, the abundance of the miRNA novel_390 was increased by exposure to salinity stress, while the abundance of novel_246 was reduced; three targets of these miRNAs are genes associated with JA signaling: one encodes AOC and the other two are TIFY transcription factors. The implication is that JA signaling may contribute significantly to salinity tolerance.

### Carbohydrate metabolism is an important determinant of salinity tolerance

The stress imposed by salinity exerts a combined osmotic, toxicity and oxidative effect. One of the ways plants have evolved to cope with salinity stress is to accumulate compatible solutes to provide protection against osmotic stress, to stabilize dehydrated enzymes and membranes, and to reduce the damage caused by desiccation [42]. The leading compounds involved are simple sugars such as sucrose, trehalose, raffinose and fructans [43]. It has been suggested that the accumulation of trehalose can strengthen tolerance to both salinity and drought stress [44]. There is also evidence to support the notion that these soluble carbohydrates can neutralize ROS, especially when present at high concentrations [45–47]. Here, the products of the gene targets of four of the miRNAs which were accumulated in response to salinity (novel_190, novel_312, novel_374, and novel_421) all involved the synthesis of carbohydrates (trehalose, xylose, mannose and pectin), implying that carbohydrate metabolism is important for salinity tolerance.

### Proton transporting play important roles in alkalinity response

The distinctive feature of alkalinity, as compared to salinity stress is that the pH is at a level higher than what can be readily tolerated, thus the proton transport was very important for alkalinity response, which was usually achieved by actively pumping protons. Proton pumps are provided by ATPases located either in the plasma membrane (P-type H^+^-ATPase) or the vacuole (V-type H^+^-ATPase), and by proton pyrophosphatases (H^+^-PPases). A P-type H^+^-ATPase AHA2, the activity of which was regulated by PKS5 (a serine/threonine protein kinase) and J3 (a chaperone), could transport proton out of the plasma membrane and thus modulate pH in the rhizosphere directly [4, 5]. Meanwhile, V-type H^+^-ATPase was also associated with alkalinity response. The transcript levels and activity of V-type H^+^-ATPase were induced by saline-alkali treatments, while overexpression of H^+^-ATPase could enhance tolerance of alfalfa to saline-alkali stress [48, 49]. V-type H^+^-ATPase was reported to have positive correlation with saline and osmotic tolerance [50, 51], as the raised proton gradient by it could promote the sequestration of Na^+^ into the vacuole through Na^+^/H^+^ exchanger [52]. However, the mechanism of its function in alkali stress response was still not very clear, except for some items indicated ROS metabolism and proline synthesis might be involved in this process [49]. A number of genes encoding proton pumps have been shown to be induced by alkalinity stress, and some of these are more abundantly transcribed in SR4 than in JN177 [14]. Here, the abundance of tae-miR9666b was reduced in alkalinity- stressed SR4 plants. As one of its targets, V-type H^+^-ATPase might be up-regulated and have important function for the alkalinity tolerance of SR4. The implication is that the differential transcription of proton pumps may enable SR4 to tolerate alkalinity stress more effectively.

## Conclusions

The stress imposed by salinity is distinct from that imposed by alkalinity, although the two stresses frequently occur together. It has been possible to select for an improved response to both stresses among the derivatives of an asymmetric somatic hybrid between bread wheat and tall wheatgrass: while SR3 performs better than JN177 in the face of salinity stress, SR4 is superior with respect to alkalinity tolerance. Here, the close genetic relationship between JN177, SR3 and SR4 was exploited to determine the contribution miRNAs make to abiotic stress tolerance. The data imply that auxin signaling and epigenetic regulation are both important for the expression of both tolerances; meanwhile JA signaling and carbohydrate metabolism may be more important for salinity tolerance, and proton pumping is vital for alkalinity tolerance.

## Methods

### Plant materials and growing conditions

Both SR3 and SR4 are derivatives of an asymmetric somatic hybrid between the bread wheat cultivar JN177 and tall wheatgrass (*Thinopyrum ponticum*) [12]. To obtain the RNA used for miRNA and degradome sequencing, as well as for physiological analysis, JN177, SR3 and SR4 grains were germinated on moist filter paper for two days at 23 °C and then transferred to half strength Hoagland’s liquid medium under a 16 h photoperiod, 50% relative humidity and 300 μmol m^− 2^ s^− 1^ photon flux density. Salinity and alkalinity stress was applied at the ninth day after sowing (DAS, about two-leaf-stage) by adding either 200 mM NaCl or 100 mM mixed salts (NaHCO_3_:Na_2_CO_3_ in a molar ratio of 9:1) [14, 37]. The solution was changed daily. RNA was extracted from snap-frozen roots of seedlings exposed to stress for 24 h. Meanwhile, the seedlings in the stress treatment and control were harvested for the measurement of ROS and leaf electrical conductivity. Each measurement represented the mean of ten seedlings, and each treatment/genotype combination was replicated three times. To compare the tolerance of the three lines over an extended period of stress, they were planted in a soil-filled containers (4 m × 5 m × 1 m) which were irrigated with either fresh water, 0.4% NaCl (about 68.4 mM) or 60 mM mixed alkali salts (NaHCO_3_:Na_2_CO_3_ in a molar ratio of 9:1). The whole experiment was run in triplicate, with each container planted with three 4 m rows of each of JN177, SR3 and SR4 (45 g grain per line), spaced 25 cm apart. The pH of the soil was monitored once a week from 150 to 180 DAS.

### Physiological indicators of stress

The electrical conductivity of leaves was measured using a conductivity meter. 1 g wheat leaves were took into 10 mL redistilled water, vacuumed for 20 min and immersed for another 20 min at room temperature. Then the electrical conductivity of the liquor was measured. The leaves were then boiled for 10 min and cooled, after which the conductivity of the liquor was re-measured. A relative conductivity value was calculated from the expression (conductivity after immersion)/(conductivity after boiling). Tissue H_2_O_2_ content was assessed using a method based on 3,3′-diaminobenzidine (DAB) staining, as described by Thordal-Christensen et al. [53]. It was also measured using an H_2_O_2_ colorimetric assay kit (Beyotime, Shanghai, China), in which a 0.1 g aliquot of root tissue was snap-frozen and ground to a powder, suspended in 2 mL lysis solution and centrifuged (12,000 *g*, 4 °C, 5 min); a 50 μL aliquot of the supernatant was mixed with 100 μL of the H_2_O_2_ detection solution and the absorbance of the solution measured at 560 nm, and converted into a concentration using a standard curve. The superoxide content of root tissue was measured following the method given by Jones et al. [54]. The root tip samples were immersed for 1 h in phosphate buffered saline containing 0.5 mg/mL nitroblue tetrazolium, after which the reaction was stopped by boiling the root in 80% *v*/v methanol for 10 min. The relative superoxide content of each sample was derived from photographic images by estimating the mean pixel density across the roots.

### Small RNA and degradome library sequencing

Root tissues from 9 samples (JN177, SR3, SR4 seedlings of two leaves stage under no stress, salt stress or alkali stress for 24 h) were grinded and the total RNA was extracted using the TRIzol reagent (Invitrogen, California, USA), following the recommended protocol. For the purpose of constructing small RNA libraries, the extracted RNA was first electrophoretically separated through a 15% polyacrylamide gel to allow fragments in the length range 18–30 nt to be purified. These were ligated to an RNA adapter using T_4_ RNA ligase. After reverse transcription and PCR amplification, the resulting DNA was sequenced on an Illumina HiSeq 2000 device (Novogene, Beijing, China).

For the purpose of degradome sequencing, 9 samples as described above were prepared and equal mass of root tissue from JN177 under no stress, salt stress or alkali stress were harvested and mixed to build one library, while equal mass of root tissue from the other six samples were mixed to be another library. Total RNA was extracted by TRIzol method and mRNAs with 5′-monophosphates were ligated to a 5′ adaptor, followed by reverse transcription and PCR. The construction of degradome library was according to Gao et al. [55]. Degradome sequencing was performed on a HiSeq 2000 device (BGI, Shenzhen, China). The raw sequence data of small RNA library and degradome library were respectively deposited in NCBI Sequence Read Archive (SRA, www.ncbi.nlm.nih.gov/sra) with accession number PRJNA420197 and PRJNA420207.

### Analysis of sequence data

Raw reads from the small RNA libraries were initially edited with Fastx-toolkit pipeline (http://hannonlab.cshl.edu/fastx_toolkit/) to remove adapter sequences, low quality reads, repetitive reads and reads longer than 30 nt or shorter than 18 nt. The resulting reads were then aligned against both a wheat expressed sequence tag database (ftp.ncbi.nih.gov/repository/UniGene/Triticum_aestivum/Ta.seq.uniq.gz) and a genomic sequence database (ftp://ftpmips.helmholtz-muenchen.de/plants/wheat/IWGSC/genePrediction_v2.2/) with Bowtie. The set of perfectly matching reads was queried against the Rfam database (rfam.xfam.org/) in order to remove rRNA, tRNA, snRNA and snoRNA sequences. The residual reads were then subjected to a BLAST analysis against miRBase v21 (www.mirbase.org), and those sequences showing no mismatches were considered as “known” miRNAs. Potential novel miRNAs were identified from the remaining reads by using miREvo [15] and mirdeep2 [16] to predict miRNA precursors. Sequences mapping to a precursor forming a hairpin structure were designated as “novel” miRNAs. The abundance of miRNAs in the various libraries was normalized to transcripts per million (TPM). Differentially abundant miRNAs, were called based on a significance threshold of 0.05 (false discovery rate threshold of 0.001) and a log2 ratio threshold of 1. Only miRNAs with an abundance > 10 TPM in at least one of the libraries were used to test for differential abundance between libraries. For the degradome analysis, raw reads were initially filtered to remove adapters and low quality reads, and the remaining reads were then aligned with wheat expressed sequence to reveal sliced miRNA targets, with the help of the CleaveLand 3.0 pipeline [56]. Alignments scoring up to 5 with no mismatches at the cleavage site (nucleotides 10 and 11) were considered as potential targets. The prediction of targets was obtained by applying psRNATarget software [57] against wheat expressed sequence.

### qRT-PCR analysis

The template used for the quantification of individual miRNAs was a 2 μg aliquot of DNase-treated total RNA reverse-transcribed using an miRNA cDNA kit (TIANGEN Biotech, Beijing, China). qRT-PCRs were formulated using an miRNA Real-Time PCR Assay kit (TIANGEN Biotech, Beijing, China). To estimate the abundance of target transcripts, a 2 μg aliquot of DNase-treated total RNA was reverse-transcribed using FastKing RT kit (TIANGEN Biotech, Beijing, China), following the manufacturer’s protocol. All qRT-PCRs were performed using an iCycler iQTM real-time PCR detection system (Bio-Rad, Hercules, California, USA). The wheat gene *U6* (GenBank: X63066.1) was chosen as the reference for the miRNA reactions and *cyclophilin A* (GenBank: AY456123.1) for the mRNAs reactions [58, 59]. The tobacco gene *L25* (GenBank: L18908.1) was used as reference for expression analysis of GUS [60]. Abundances were normalized using the 2^-ΔΔCt^ method. Each assay was represented by three biological replicates, each of which was calculated from the mean of three technical replicates.

### Validation of miRNA targets by transient expression in tobacco

Mature miRNAs, targets and mutated target fragments were respectively cloned into the pStart-GUS vector [61] driven by the 35S promoter, then mixed and transformed into tobacco leaves using *Agrobacterium tumefaciens* as the vector [62]. After culturing for three days (one day in the dark and two 16 h photoperiod days under 300 μmol m^− 2^ s^− 1^ photon flux density), the leaves were harvested for histochemical GUS staining following Jefferson et al. [63]. Each assay was represented by three biological replicates. The relevant primer sequences are shown in Additional file 22: Table S14.

For RLM 5’-RACE, total RNA of tobacco leaves co-expressed miRNA and corresponding target were extracted by TRIzol and reverse-transcribed using an miRNA cDNA kit with a random primer (TIANGEN Biotech, Beijing, China). The reverse-transcript product was treated by RNase H and then purified by PCR clean up kit (Beyotime, Shanghai, China). The cDNA was then added poly C by Terminal Deoxynucleotidyl Transferase (Thermo Scientific, USA) with dCTP and then the purified product could be used as template for PCR. An AUP primer was used for 8 cycles PCR as the denaturing temperature was 40 °C,and then a touch-down PCR were used with primer NUP and GSP. The PCR product was cloned and sequenced to identify the cleavage site.

### VIGS analysis

The required pri-miRNAs were cloned into the pEasy-blunt vector (Transgen Biotech, Beijing, China) using the primers listed in Table S14. After sequencing, the appropriate fragments were ligated to the γ vector, then co-expressed in tobacco leaves together with α and β vectors by *Agrobacterium*. After culturing for three days (one day in the dark and two 16 h photoperiod days under 300 μmol m^− 2^ s^− 1^ photon flux density), ~ 5 g leaf was homogenized in 20 mM sodium phosphate (pH 7.2) containing 1% *w*/*v* celite. The paste was applied to wheat seedling leaves, and the abundance of the specific miRNA was estimated after two weeks. The seedlings were subsequently exposed to either 200 mM NaCl or 100 mM mixed alkali salts (NaHCO_3_:Na_2_CO_3_ in a molar ratio of 9:1) for a further week to assess their stress response.

## Additional files

Additional file 1: **Figure S1.** ROS content in the tissue of SR3/SR4 and JN177 in response to exposure to stress. **(A)** The nitroblue tetrazolium assay for superoxide in the root tips of JN177, SR3 and SR4 grown under control (CK), saline (NaCl) or alkaline (pH 8.9) conditions. **(B)** The superoxide content of the above samples normalized to the superoxide content of JN177 roots raised under non-stressful conditions. **(C)** The DAB assay for H_2_O_2_ in the root tips of plants grown under control, saline or alkaline conditions. **(D)** The H_2_O_2_ content of the above samples normalized to the H_2_O_2_ content of JN177 roots raised under non-stressful conditions. Data given in the form mean ± s.d. (*n* = 3). *, **: means differed significantly at, respectively, *P* < 0.05 and < 0.01, as determined by the Student’s *t*-test. (TIF 10622 kb)

Additional file 2: **Figure S2.** The length distribution of small RNAs (18–30 nt). Small RNAs extracted from JN177, SR3 and SR4 plants grown **(A)** under non-stressed conditions, **(B)** in the presence of salinity stress, **(C)** in the presence of alkalinity stress. (TIF 716 kb)

Additional file 3: **Figure S3.** Categorization of small RNAs. Clean reads obtained from the nine libraries were mapped onto wheat genome sequence, and the “+”mapped small RNAs assigned to the various categories of small RNA. The proportion of “known” and “novel” miRNAs is shown in the form of pie charts. JNC, SR3C, SR4C: JN177, SR3, SR4 seedlings grown under non-stressed conditions; JNS, SR3S, SR4S: JN177, SR3, SR4 seedlings stressed by salinity; JNA, SR3A, SR4A: JN177, SR3, SR4 seedlings stressed by alkalinity. (TIF 1527 kb)

Additional file 4: **Table S1.** The 49 “known” miRNAs, belonging to 27 families. (DOCX 13 kb)

Additional file 5: **Table S2.** The mature and pre-sequences of 85 “known” miRNAs. (XLSX 17 kb)

Additional file 6: **Table S3.** The mature and pre-sequences of 219 “novel” miRNAs. (XLSX 25 kb)

Additional file 7: **Table S4.** The “novel” miRNAs with homologs in other species. (DOCX 13 kb)

Additional file 8: **Table S5.** The abundance of “novel” miRNAs. (XLSX 50 kb)

Additional file 9: **Figure S4.** Bias at the first base in miRNAs. The relative frequency of A (red), T (blue), C (green) and G (brown) nucleotides occurring as the first base of both known and novel miRNAs. JN: JN177; the “C” in JNC, SR3C, SR4C refers to seedlings grown under non-stressed conditions, while the “S” and “A” suffix refers to seedlings grown in the presence of, respectively, salinity and alkalinity. The number shown above each column represents the number of reads of equal length. (TIF 2756 kb)

Additional file 10: **Figure S5.** Base bias across the miRNA sequences. Color coding as in Fig. S4. (TIF 4192 kb)

Additional file 11: **Table S6.** The set of salinity and/or alkalinity responsive miRNAs in JN177. (XLSX 16 kb)

Additional file 12: **Table S7.** The set of differentially abundant miRNAs between JN177 and either SR3 or SR4. (XLSX 22 kb)

Additional file 13: **Table S8.** The predicted targets of the set of miRNAs. (XLSX 40 kb)

Additional file 14: **Table S9.** The targets of miRNAs as identified by degradome sequencing. (XLSX 31 kb)

Additional file 15: **Figure S6.** Mapping the target mRNA cleavage sites for five miRNAs. Three miRNA/target pairs (novel_246/*traes_2DL_339870EAD.1,* novel_27/*traes_7BL_7E8BDC838.1* and novel_84/*traes_1BL_63019C378.1*) were confirmed by degradome analysis. Two miRNA/target pairs (miR1120c/*traes_2BS_5C64FC44A.2* and miR9664/*traes_2BS_5045C640C.2*) were predicted by psRNATarget software. (TIF 1013 kb)

Additional file 16: **Figure S7.** GO analysis of the target genes of miRNAs which were altered in abundance in response to abiotic stress. The target genes were identified by degradome sequencing. MF: molecular function, BP: biological process. The targets of miRNAs which were altered in abundance in response to **(A)** salinity stress, **(B)** alkalinity stress. (TIF 2668 kb)

Additional file 17: **Table S10.** Pathways significantly enriched under saline or alkaline stress, as determined by KEGG analysis. (XLSX 12 kb)

Additional file 18: **Table S11.** The targets in JN177 of the saline and alkaline responsive miRNAs. (XLSX 26 kb)

Additional file 19: **Table S12.** The targets of the miRNAs associated with salinity tolerance. (XLSX 13 kb)

Additional file 20: **Table S13.** The targets of the miRNAs associated with alkalinity tolerance. (XLSX 12 kb)

Additional file 21: **Figure S8.** The abundance of miR1120c and miR9664 in SR3/SR4 and JN177 exposed to stress**.** miRNA abundance in plants experiencing **(A,C)** salinity stress, **(B,D)** alkalinity stress. qRT-PCR outputs are given in the form mean ± s.d. (*n* = 3). *, **: means differed significantly at, respectively, *P* < 0.05 and < 0.01, as determined by the Student’s *t*-test. (TIF 643 kb)

Additional file 22: **Table S14.** Primer sequences used in the study. (XLSX 12 kb)

## Abbreviations

AOC: allene oxide cyclase
JAZ: jasmonate ZIM domain-containing protein
MYB: myeloblastosis
NAC: NAM, ATAF1,2, CUC2
NBT: Nitro blue tetrazolium
POD: Peroxidase
qRT-PCR: quantitative real-time PCR
RLK: receptor like kinase
ROS: reactive oxygen species
SOD: superoxide dismutase
SUMO: small ubiquitin-related modifier
TPM: transcripts per million
