# Supplementary figures and images for: Small RNA and degradome sequencing used to elucidate the basis of tolerance to salinity and alkalinity in wheat
Source: BMC Plant Biol. 2018 Sep 15;18:195. doi: 10.1186/s12870-018-1415-1 (PMC6139162; doi:10.1186/s12870-018-1415-1)

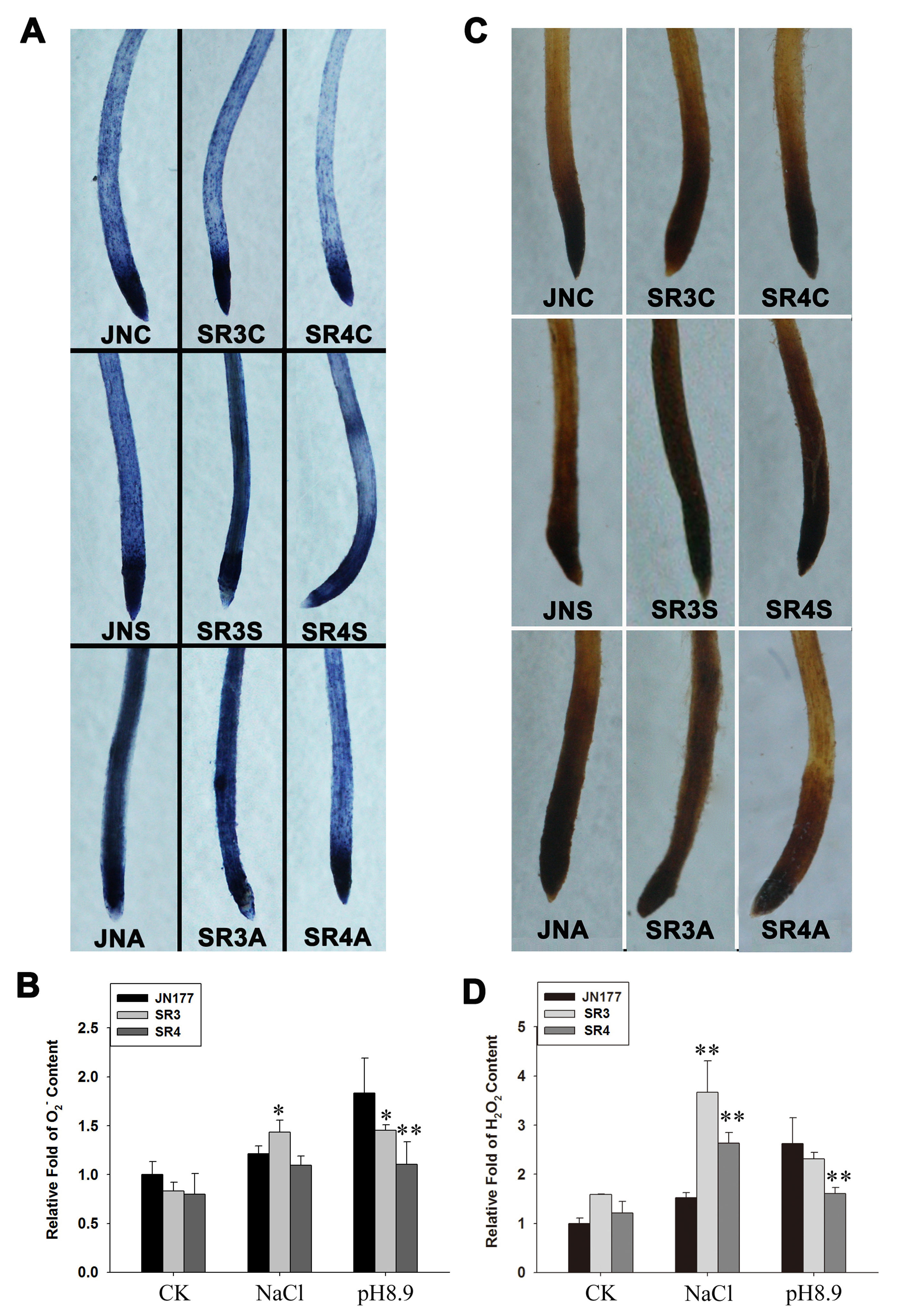

Supplement: Supplementary file 1 — Figure S1. ROS content in the tissue of SR3/SR4 and JN177 in response to exposure to stress. (A) The nitroblue tetrazolium assay for superoxide in the root tips of JN177, SR3 and SR4 grown under control (CK), saline (NaCl) or alkaline (pH 8.9) conditions. (B) The superoxide content of the above samples normalized to the superoxide content of JN177 roots raised under non-stressful conditions. (C) The DAB assay for H2O2 in the root tips of plants grown under control, saline or alkaline conditions. (D) The H2O2 content of the above samples normalized to the H2O2 content of JN177 roots raised under non-stressful conditions. Data given in the form mean ± s.d. (n = 3). *, **: means differed significantly at, respectively, P < 0.05 and < 0.01, as determined by the Student’s t-test. (TIF 10622 kb) [file 12870_2018_1415_MOESM1_ESM.tif]

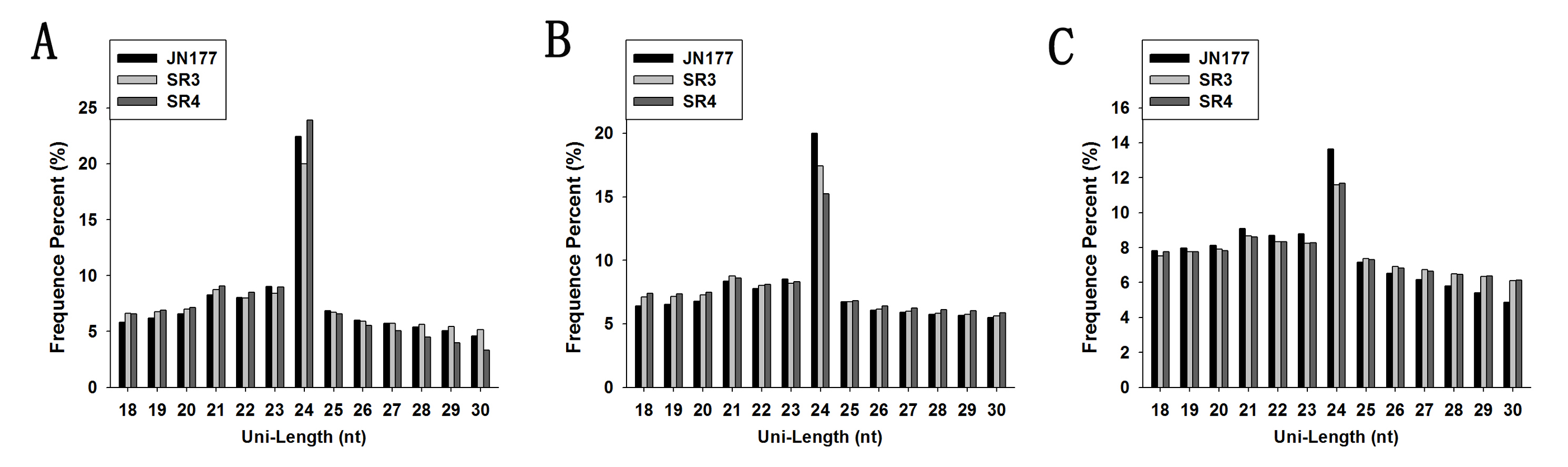

Supplement: Supplementary file 2 — Figure S2. The length distribution of small RNAs (18–30 nt). Small RNAs extracted from JN177, SR3 and SR4 plants grown (A) under non-stressed conditions, (B) in the presence of salinity stress, (C) in the presence of alkalinity stress. (TIF 716 kb) [file 12870_2018_1415_MOESM2_ESM.tif]

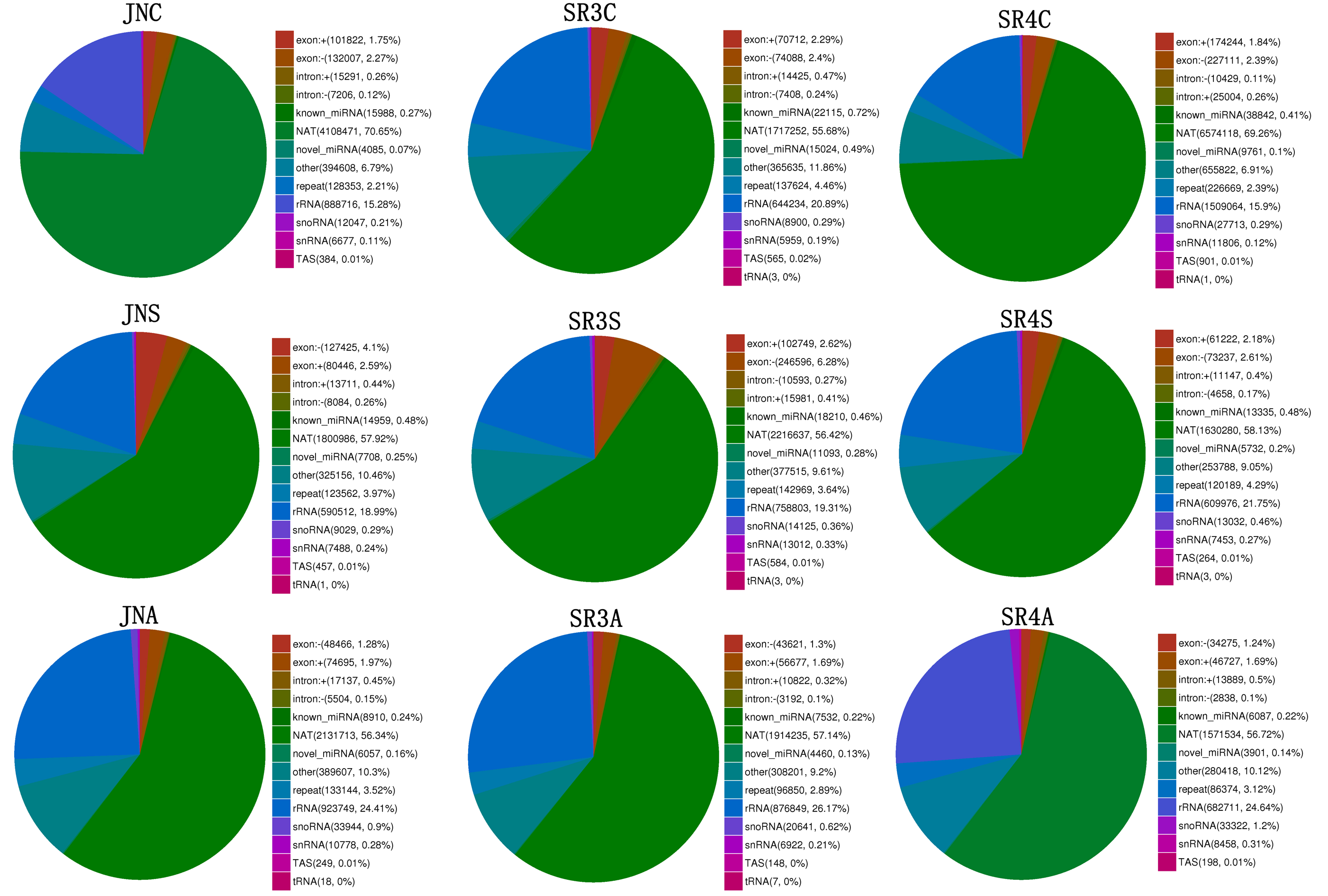

Supplement: Supplementary file 3 — Figure S3. Categorization of small RNAs. Clean reads obtained from the nine libraries were mapped onto wheat genome sequence, and the “+”mapped small RNAs assigned to the various categories of small RNA. The proportion of “known” and “novel” miRNAs is shown in the form of pie charts. JNC, SR3C, SR4C: JN177, SR3, SR4 seedlings grown under non-stressed conditions; JNS, SR3S, SR4S: JN177, SR3, SR4 seedlings stressed by salinity; JNA, SR3A, SR4A: JN177, SR3, SR4 seedlings stressed by alkalinity. (TIF 1527 kb) [file 12870_2018_1415_MOESM3_ESM.tif]

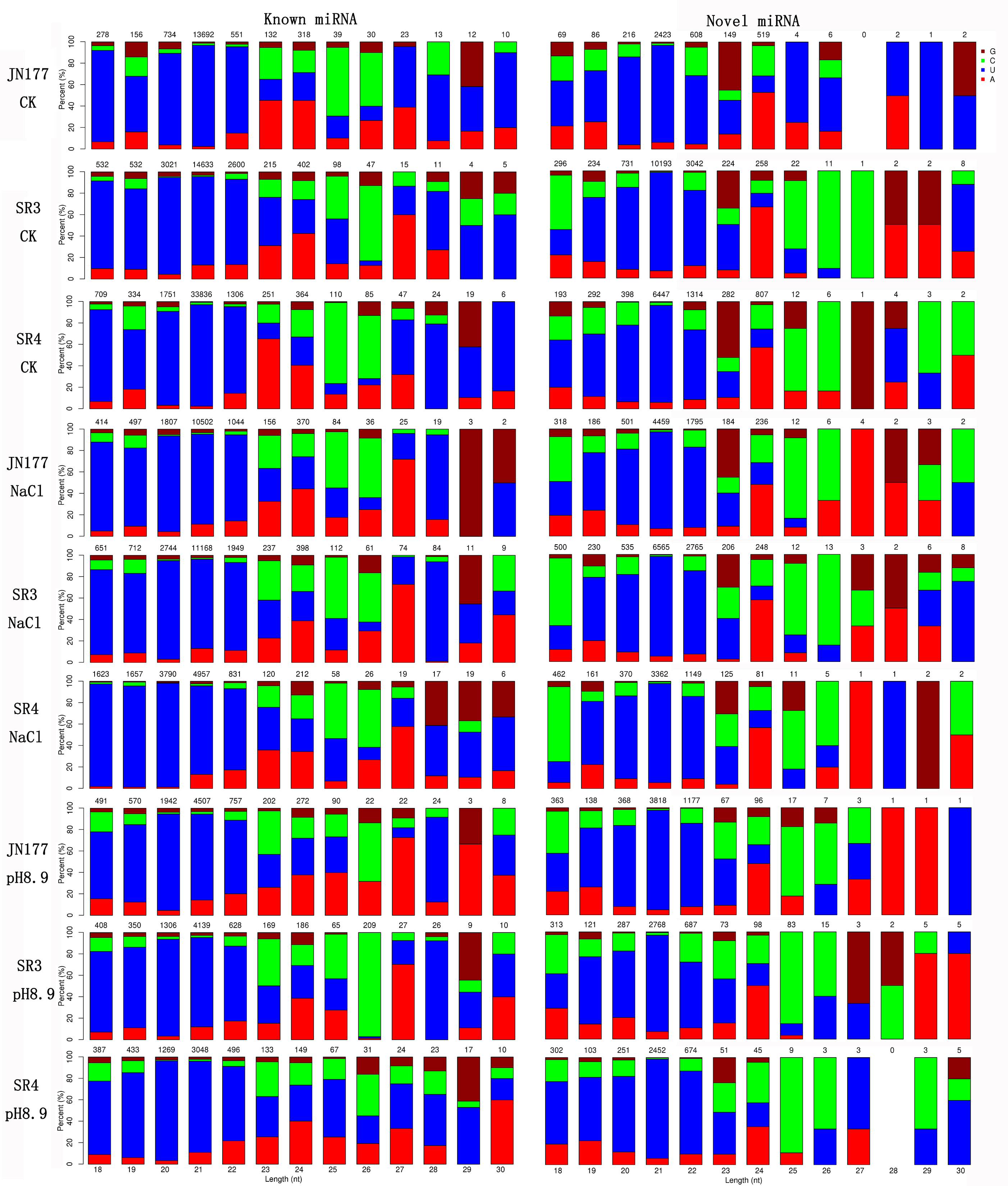

Supplement: Supplementary file 9 — Figure S4. Bias at the first base in miRNAs. The relative frequency of A (red), T (blue), C (green) and G (brown) nucleotides occurring as the first base of both known and novel miRNAs. JN: JN177; the “C” in JNC, SR3C, SR4C refers to seedlings grown under non-stressed conditions, while the “S” and “A” suffix refers to seedlings grown in the presence of, respectively, salinity and alkalinity. The number shown above each column represents the number of reads of equal length. (TIF 2756 kb) [file 12870_2018_1415_MOESM9_ESM.tif]

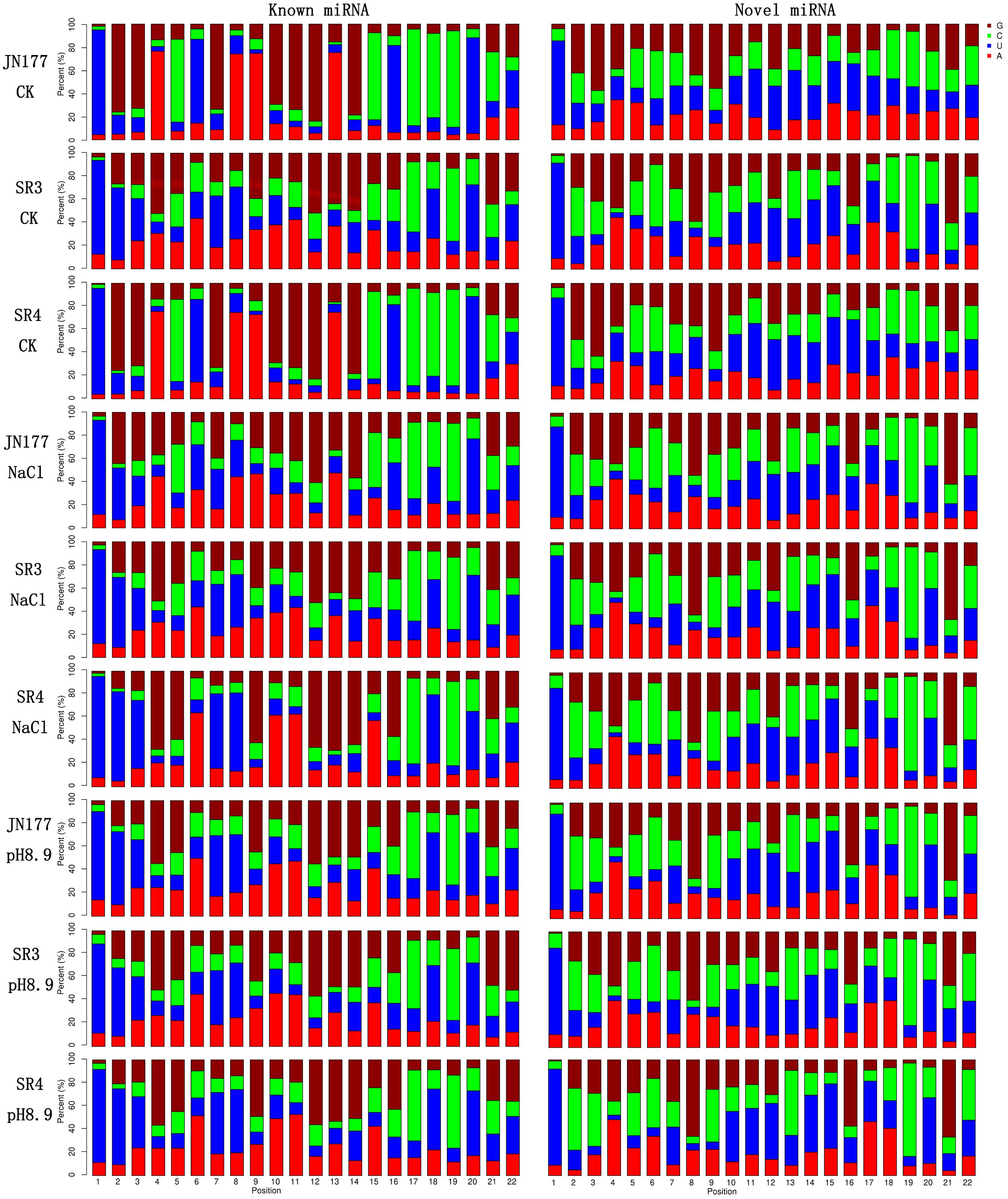

Supplement: Supplementary file 10 — Figure S5. Base bias across the miRNA sequences. Color coding as in Fig. S4. (TIF 4192 kb) [file 12870_2018_1415_MOESM10_ESM.tif]

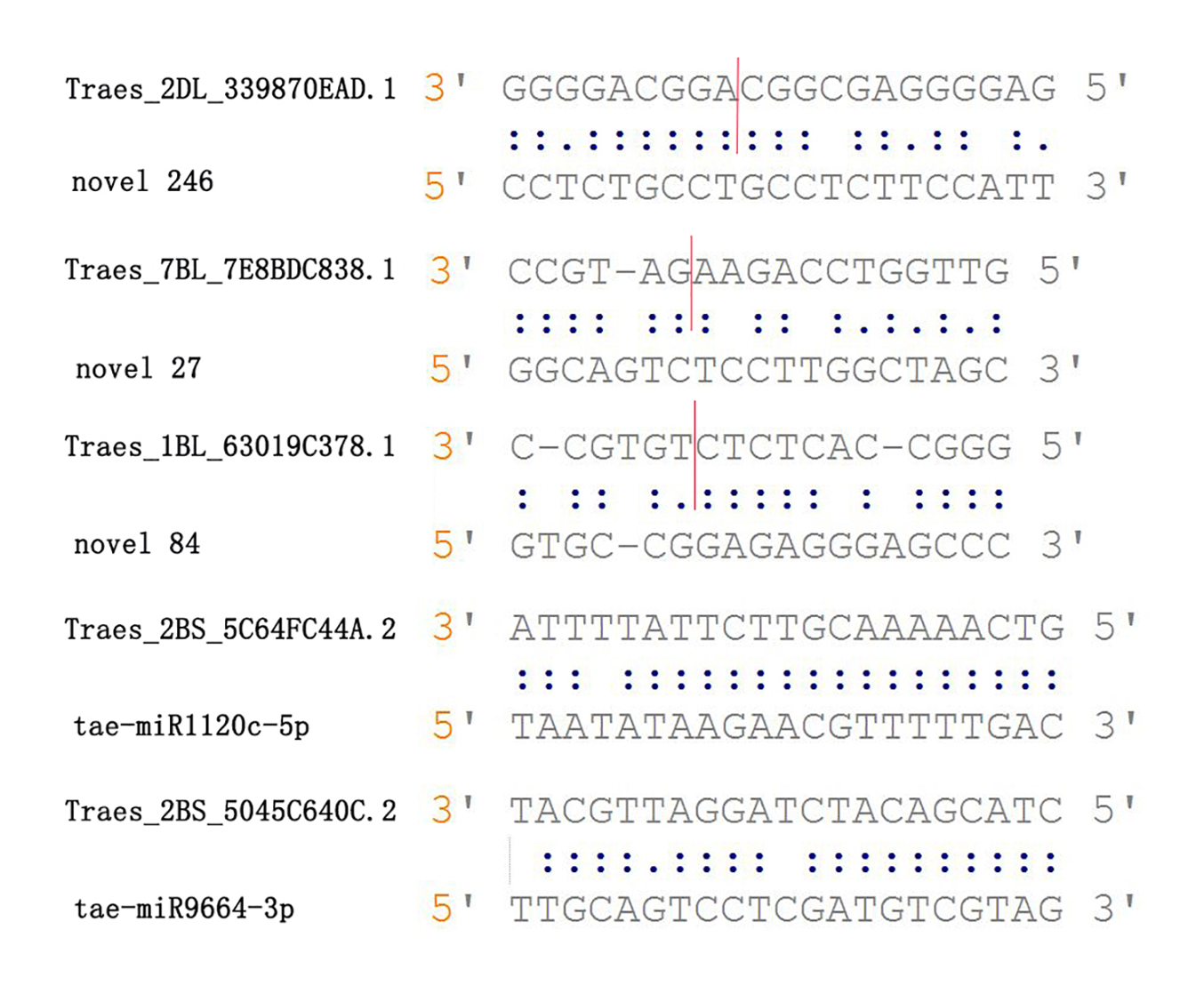

Supplement: Supplementary file 15 — Figure S6. Mapping the target mRNA cleavage sites for five miRNAs. Three miRNA/target pairs (novel_246/traes_2DL_339870EAD.1, novel_27/traes_7BL_7E8BDC838.1 and novel_84/traes_1BL_63019C378.1) were confirmed by degradome analysis. Two miRNA/target pairs (miR1120c/traes_2BS_5C64FC44A.2 and miR9664/traes_2BS_5045C640C.2) were predicted by psRNATarget software. (TIF 1013 kb) [file 12870_2018_1415_MOESM15_ESM.tif]

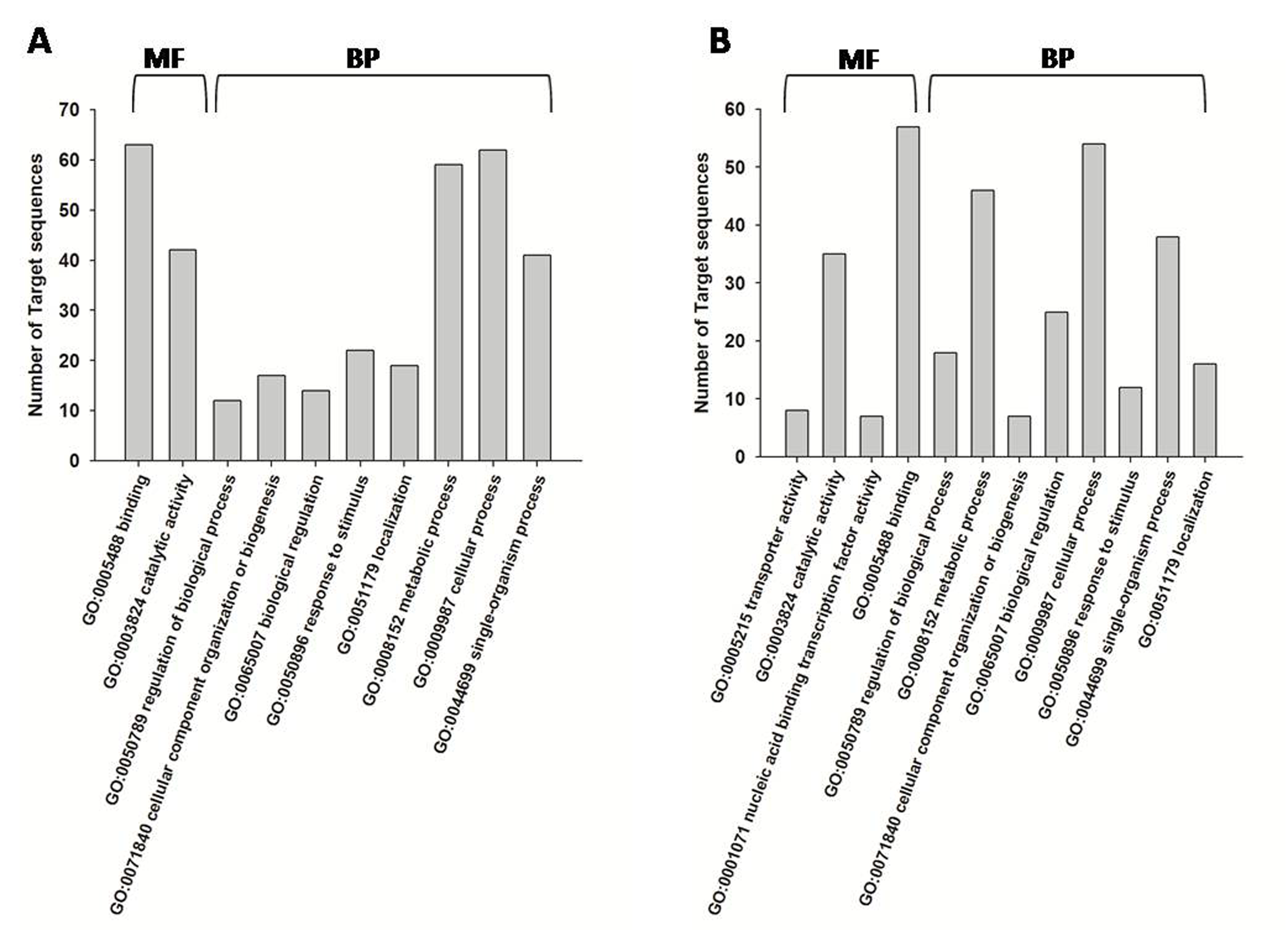

Supplement: Supplementary file 16 — Figure S7. GO analysis of the target genes of miRNAs which were altered in abundance in response to abiotic stress. The target genes were identified by degradome sequencing. MF: molecular function, BP: biological process. The targets of miRNAs which were altered in abundance in response to (A) salinity stress, (B) alkalinity stress. (TIF 2668 kb) [file 12870_2018_1415_MOESM16_ESM.tif]

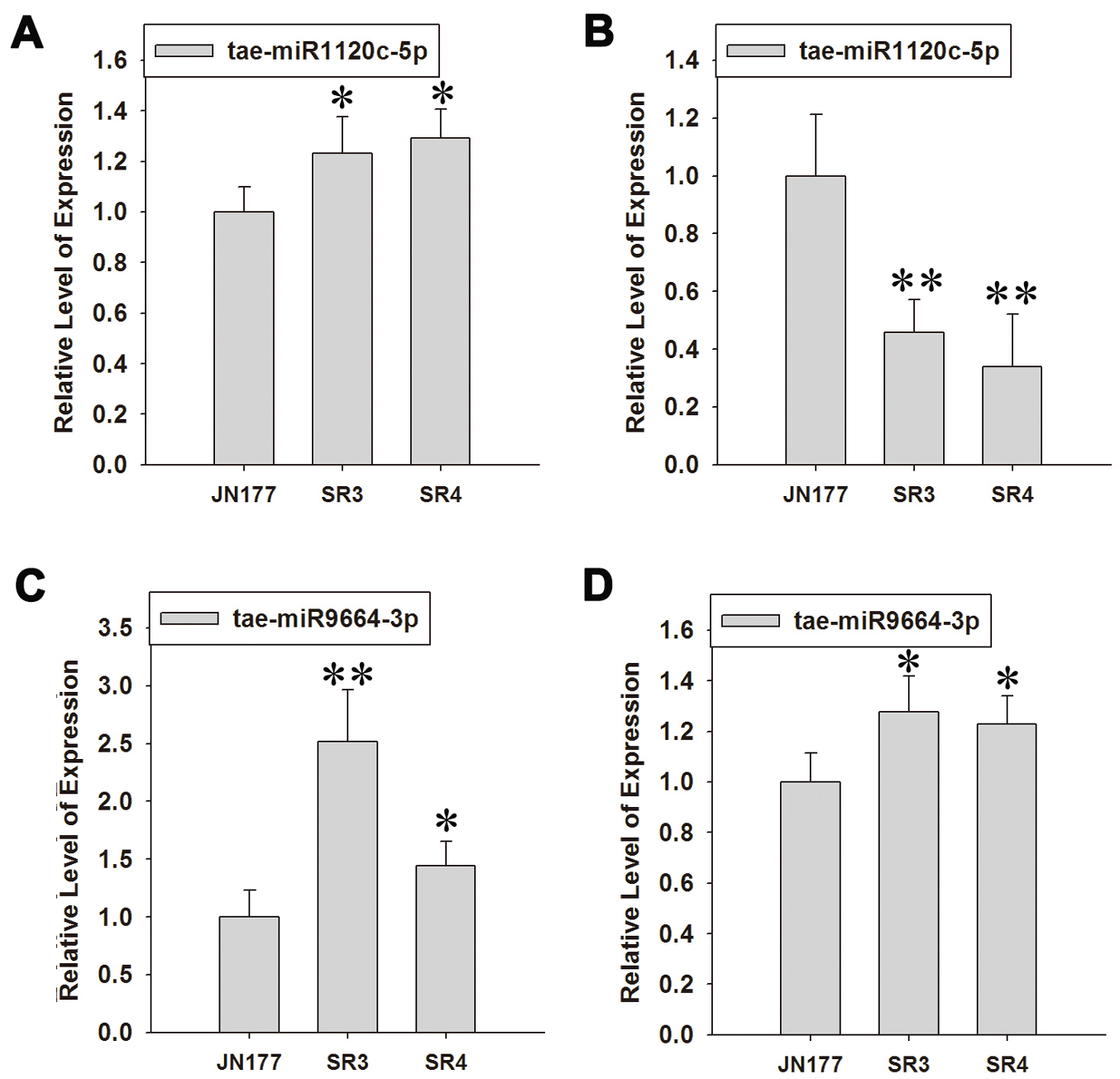

Supplement: Supplementary file 21 — Figure S8. The abundance of miR1120c and miR9664 in SR3/SR4 and JN177 exposed to stress. miRNA abundance in plants experiencing (A,C) salinity stress, (B,D) alkalinity stress. qRT-PCR outputs are given in the form mean ± s.d. (n = 3). *, **: means differed significantly at, respectively, P < 0.05 and < 0.01, as determined by the Student’s t-test. (TIF 643 kb) [file 12870_2018_1415_MOESM21_ESM.tif]
