# Supplementary material for: Small RNA and degradome sequencing used to elucidate the basis of tolerance to salinity and alkalinity in wheat
Source: BMC Plant Biol. 2018 Sep 15;18:195. doi: 10.1186/s12870-018-1415-1 (PMC6139162; doi:10.1186/s12870-018-1415-1)
Supplement: Supplementary file 4 — Table S1. The 49 “known” miRNAs, belonging to 27 families. (DOCX 13 kb) [file 12870_2018_1415_MOESM4_ESM.docx]

Table S1. The 49 known Tae-miRNAs belonging to 27 families.

| miRNA family | known Tae-miRNA |
| --- | --- |
| MIR444 | tae-MIR444a |
| MIR5200 | tae-MIR5200 |
| MIR398 | tae-MIR398 |
| MIR9657 | tae-MIR9657a tae-MIR9657c |
| MIR1120 | tae-MIR1120a tae-MIR1120b tae-MIR1120c tae-MIR1121 tae-MIR1122c tae-MIR1137a tae-MIR1137b |
| MIR159 | tae-MIR159a tae-MIR319 |
| MIR9674 | tae-MIR9674a tae-MIR9674b |
| MIR9662 | tae-MIR9662a tae-MIR9662b |
| MIR9666 | tae-MIR9666a tae-MIR9666b |
| MIR9672 | tae-MIR9672a tae-MIR9672b |
| MIR397 | tae-MIR397 |
| MIR408 | tae-MIR408 |
| MIR9654 | tae-MIR9654a tae-MIR9654b |
| MIR164 | tae-MIR164 |
| MIR160 | tae-MIR160 |
| MIR7757 | tae-MIR7757 |
| MIR5084 | tae-MIR5084 |
| MIR9772 | tae-MIR9772 |
| MIR171_1 | tae-MIR171a tae-MIR171b |
| MIR1122 | tae-MIR1122a tae-MIR1127a tae-MIR1127b tae-MIR1128 tae-MIR1133 tae-MIR1135 tae-MIR1136 |
| MIR5062 | tae-MIR5062 |
| MIR818 | tae-MIR1130b |
| MIR5067 | tae-MIR5049 |
| MIR156 | tae-MIR156 |
| MIR395 | tae-MIR395a tae-MIR395b |
| MIR9783 | tae-MIR9783 |
| MIR167_1 | tae-MIR167a tae-MIR167c |
