# Supplementary material for: Small RNA and degradome sequencing used to elucidate the basis of tolerance to salinity and alkalinity in wheat
Source: BMC Plant Biol. 2018 Sep 15;18:195. doi: 10.1186/s12870-018-1415-1 (PMC6139162; doi:10.1186/s12870-018-1415-1)
Supplement: Supplementary file 7 — Table S4. The “novel” miRNAs with homologs in other species. (DOCX 13 kb) [file 12870_2018_1415_MOESM7_ESM.docx]

Table S4 The novel wheat miRNAs homologous to known miRNA in other species

| known miRNA in miRbase | novel miRNA in wheat |
| --- | --- |
| MIR1432 | novel_223 |
| MIR156 | novel_239,novel_352 |
| MIR159 | novel_234 |
| MIR160 | novel_53 |
| MIR164 | novel_231,novel_375 |
| MIR166 | novel_1,novel_364,novel_336 |
| MIR167 | novel_199,novel_227,novel_64 |
| MIR169 | novel_225 |
| MIR171a | novel_10 |
| MIR172 | novel_262 |
| MIR319 | novel_2,novel_205 |
| MIR390 | novel_248 |
| MIR393 | novel_79 |
| MIR396 | novel_38,novel_196,novel_307 |
| MIR398 | novel_186,novel_22 |
| MIR399 | novel_267 |
| MIR528 | novel_246 |
| MIR530 | novel_252 |
| MIR531 | novel_274 |
| MIR9652 | novel_285 |
| MIR9661 | novel_362 |
| MIR9676 | novel_112 |
| MIR9772 | novel_204,novel_214 |
| MIR9776 | novel_182 |
